# Supporting Patients With Breast Cancer and Providers Through Treatment and Survivorship: Multimethod Implementation Study of the MyJourney Platform

**DOI:** 10.2196/87973

**Published:** 2026-06-10

**Authors:** Isabella Herrington, Julie Makarski, Krystle Amog, Leigh Hayden, Kate Mossman, Norna Abbo, Nicole Jedrzejko, Fahima Osman, Monika Kastner

**Affiliations:** 1 Research and Innovation, North York General Hospital, 4001 Leslie Street, Toronto, ON, M2K 1E1, Canada, 1 416 756 6000; 2 Institute of Health Policy, Management and Evaluation, University of Toronto, Toronto, ON, Canada; 3 Institute of Biomedical Engineering, University of Toronto, Toronto, ON, Canada; 4 Division of General Surgery, University of British Columbia, Vancouver, BC, Canada; 5 Department of Surgery, North York General Hospital, Toronto, ON, Canada; 6 Department of Surgery, University of Toronto, Toronto, ON, Canada; 7 Department of Family and Community Medicine, University of Toronto, Toronto, ON, Canada

**Keywords:** breast cancer, qualitative methods, usability testing, implementation, patient journey

## Abstract

**Background:**

Breast cancer is the most common cancer among Canadian women, bringing complex demands for timely decision-making, coordination of multidisciplinary care, and efficient communication between patients and providers. The increasing reliance on fragmented and noninteroperable health information systems exacerbates workflow and documentation burdens, leading to inefficiencies and gaps in continuity of care. While nurse navigation programs partially bridge these gaps, most digital platforms remain poorly integrated into provider workflows, requiring manual tracking, which results in duplicated effort and reduced efficiency. Our team developed “MyJourney” at North York General Hospital (NYGH) in Ontario. It is a digital navigation platform that supports breast cancer care throughout the entire continuum, from diagnosis to survivorship.

**Objective:**

This study aimed (1) to map the breast cancer journey and workflow to inform the design and adaptation of MyJourney at 2 oncology settings at NYGH; (2) to identify barriers and guide local implementation; and (3) to evaluate the implementation, usability, and perceived utility of MyJourney’s Clinical Navigation Tool for breast cancer care teams.

**Methods:**

A multimethod, 3-phase study was conducted at NYGH’s Breast Diagnostic Centre (BDC) and Chemotherapy Clinic (CC) in Toronto, Canada. Phase 1 involved qualitative interviews with patients with breast cancer to map their care journey and inform user-centered platform design. Phase 2 included preimplementation interviews with nurses, pharmacists, and administrative staff to map workflows and customize MyJourney for the CC. Phase 3 evaluated MyJourney’s implementation and usability over 6 weeks using the System Usability Scale, technology acceptance model surveys, and follow-up interviews with platform users. Data were analyzed via interpretive description and descriptive statistics. Ethics approval was obtained.

**Results:**

In total, 13 patient interviews revealed distinct challenges and communication needs across prediagnosis, diagnosis, treatment, and survivorship phases, emphasizing the need for personalized, integrated resources (phase 1). A total of 8 participants (3 nurses and 5 pharmacists), providers, and clinic staff highlighted pain points in fragmented information systems, inefficient manual processes, and limited team coordination (phase 2). MyJourney’s phased implementation led to high user acceptance, with mean System Usability Scale scores rated “excellent” (BDC: 81.3; CC: 86.3) at the 6-week follow-up (phase 3). The 4 participants (1 charge nurse, 1 administrative staff at the BDC, and 2 charge nurses at the CC) described the platform as intuitive, efficient, and well-organized, citing consolidated patient records, streamlined appointment management, and improved workflow as major benefits. Recommendations included improved interoperability, enhanced notifications, role-specific customization, and integration with electronic medical records for broader scalability.

**Conclusions:**

The iterative, interest-holder engaged design and phased implementation of MyJourney facilitated rapid uptake and high usability among breast cancer provider teams. The Clinical Navigation Tool component of the MyJourney platform reduced documentation burden, improved workflow efficiency, and facilitated care coordination across oncology settings.

## Introduction

Breast cancer is the most common cancer in Canadian women, apart from nonmelanoma skin cancers, and is the second leading cause of cancer-related deaths [1]. Approximately 1 in 8 Canadian women will develop breast cancer during their lifetime [1,2]. Although multidisciplinary care and systemic therapies have improved breast cancer outcomes over the past decade [3], these advances have introduced new complexities for both patients and health care providers.

Patients are now expected to make timely and informed decisions, interpret detailed medical information, and coordinate care across multiple services and providers [2,4-6]. This can lead to confusion about procedures, points of contact, and treatment-related side effects, sometimes resulting in delays in care [6]. For health care providers, these complexities increase the demand for efficient documentation, coordinated communication, and real-time access to patient information [7,8]. Clinical data are often fragmented across noninteroperable health information systems, and care processes are heterogeneous and complex to manage, resulting in high levels of administrative and role-related burden [7,8].

Digital health platforms offer a promising solution to streamline care delivery, reduce manual workload, and enhance communication among health care team members [9]. However, most available platforms are not designed for provider use and are poorly integrated into clinical workflows [10]. This results in providers relying on manual tracking, which can lead to redundant documentation and time-consuming phone-based follow-up [9]. Although nurse navigation programs help bridge some of these challenges in oncology practice by supporting patients throughout their treatment journey, the model is labor-intensive [11,12]. Navigators are often required to contact patients at multiple points in their journey, without standardized tools to streamline navigation tasks or optimize workflow within and across clinical settings [11,12].

One strategy to support health care teams and reduce administrative burden is to leverage integrated digital platforms that consolidate patient care information in a single system. Digital platforms can reduce the need to search across multiple sources and allow providers to focus on direct patient care [13]. Platforms that coordinate tasks across teams can also reduce fragmentation within the health care system and enhance care continuity [13].

To address these challenges, our team developed “MyJourney,” a digital oncology platform that supports breast cancer care across the continuum, from diagnosis through survivorship. The platform was initially developed by a team of software developers and design experts for North York General Hospital (NYGH) in Ontario, Canada. Following prototype development, the platform underwent iterative refinement through a user-centered design process that engaged a broad group of interest-holders, including breast cancer providers, patients, researchers, app developers, and design experts. Interest-holder engagement began in 2017, and platform development launched in 2019. MyJourney includes three integrated components: (1) a Clinical Navigation Tool that allows care teams (including nurses, pharmacists, oncologists, and surgeons) to triage, monitor, and document patient care; (2) a Multidisciplinary Cancer Conference Tool that facilitates collaborative case planning, treatment decision-making, and reporting; and (3) a patient-facing mobile app that provides personalized treatment summaries, tailored education, appointment reminders, and real-time updates to promote engagement and self-management. The platform was designed to address dual needs. First, it streamlines workflows, reduces documentation burden, and supports team-based coordination for providers. Second, it centralizes health information, treatment plans, and appointments into one accessible location for patients diagnosed with breast cancer. These functions align with broader health system priorities, including those reflected in the Quintuple Aim, by aiming to improve both provider experience and patient outcomes and experiences [14].

The objectives of our work were (1) to understand the breast cancer journey, gaps in care, and the needs of patients at the Breast Diagnostic Centre (BDC) to inform the design of the patient-facing component of MyJourney; (2) to map workflows, identify barriers, and guide local adaptation of MyJourney before its launch at the NYGH Chemotherapy Clinic (CC); and (3) to evaluate the implementation, usability, and perceived utility of the Clinical Navigation Tool component of MyJourney at both the BDC and CC.

## Methods

### Study Design

#### Overview

**Figure 1.**
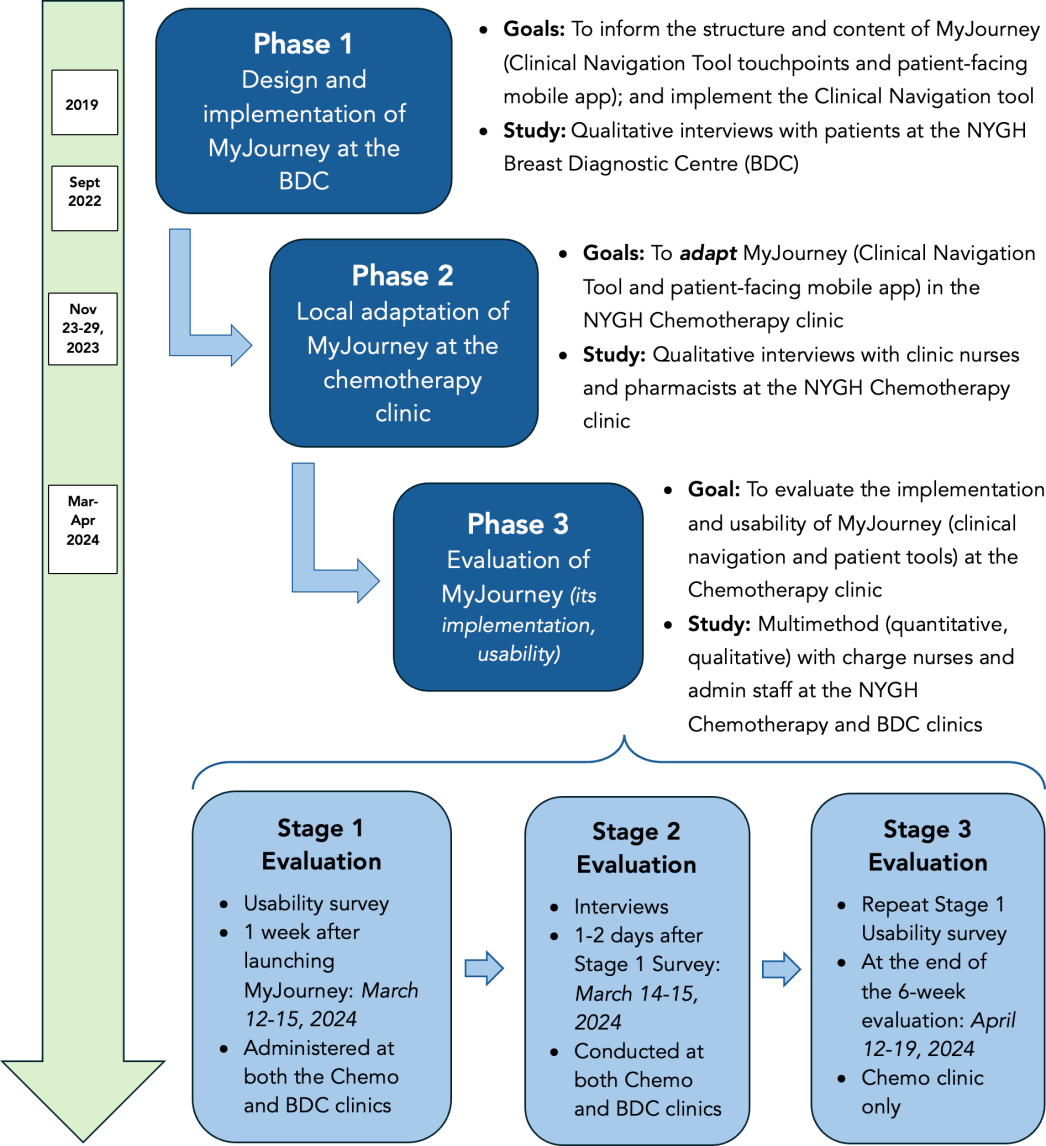
Study design schema. NYGH: North York General Hospital.

We conducted a 3-phase, multimethod study that used both qualitative and quantitative methods (Figure 1). Phase 1 patient interviews (2018‐2019) informed the initial design of MyJourney’s Clinical Navigation Tool and patient-facing app. The Clinical Navigation Tool was first implemented at the BDC in September 2022, providing a mature implementation in a diagnostic-focused setting. Lessons learned from this implementation (eg, around appointment visibility, task lists, and navigation touchpoints), together with phase 2 preimplementation workflow interviews at the CC, informed the subsequent localization and configuration of MyJourney for the treatment-focused CC in 2024. More specifically, the below sections highlight the design for each of the 3 phases.

#### Phase 1

To inform the structure and content of MyJourney (Clinical Navigation Tool touchpoints and patient-facing mobile app), we conducted qualitative interviews with patients at the NYGH BDC to map the breast cancer journey from prediagnosis through survivorship and to inform the structure and content of MyJourney (Clinical Navigation Tool and patient-facing mobile app). We used the interpretive description of Thorne [15], grounded in naturalistic inquiry and recognizing the constructed and contextual nature of human experience. It seeks applied understanding that supports practical decision-making [15] and centers on participants’ experiential accounts rather than testing or building theory, as it is grounded in practical relevance [15].

#### Phase 2

To adapt MyJourney to the NYGH CC, we conducted preimplementation interviews with staff to map workflows and staff roles, identify barriers, and tailor MyJourney’s features to support local adaptation (November 23‐29, 2023). We used the same interpretive description approach as in phase 1, which uses low-inference interpretation (ie, description) to enable the researcher to remain close to the data [15]. In this approach, facts are presented as a comprehensive summary in everyday language from the participants’ perspectives, serving both as an output of the research and as recommendations for future research [15-17]. We used qualitative results to iteratively implement the Clinical Navigation Tool component of MyJourney (December 2023-February 2024). This involved installing the MyJourney technology at the CC (integrating within their electronic medical record system) and training users (nurses and administration staff) on how to use the system. Once installed, MyJourney launched for use on March 4, 2024.

#### Phase 3

To evaluate the implementation, usability, and perceived utility of MyJourney’s Clinical Navigation Tool at the CC, we conducted a 6-week, iterative, 3-staged evaluation. One week after implementing the Clinical Navigation Tool, we administered a usability survey to MyJourney users (charge nurses and administrative staff; stage 1). After 1‐2 days, we conducted one-on-one interviews with these participants to gain a more in-depth understanding of their experiences, including workflow alignment (using survey findings as a starting point), and to identify any challenges and suggested recommendations to address them (stage 2). Over the next 5 weeks, we used survey and interview results to further refine the Clinical Navigation Tool. At 6 weeks of follow-up, we readministered the stage 1 usability survey to determine whether iterative improvements translated into enhanced usability (stage 3). We also administered the usability survey (stage 1) and interviews (stage 2) to MyJourney users at the BDC clinic to get an in-depth understanding of their user experience at that site since its implementation (September 2022). For interviews, a qualitative description approach was used to enable the researcher to remain close to the data [16]. In this approach, facts are presented as a comprehensive summary in everyday language from the participants’ perspectives, serving both as an output of the research and as recommendations for future research [16]. This overall evaluation approach was guided by the RE-AIM (Reach, Effectiveness, Adoption, Implementation, and Maintenance) implementation framework, which considers individual, organizational, and contextual factors to shape the implementation and sustainability of innovations [18]. For phase 3, RE-AIM primarily informed our focus on implementation (usability, workflow fit, and fidelity of use) and maintenance (patients’ and clinicians’ perceived likelihood of continued use and ongoing integration into routine care). Reporting of this study was guided by the SRQR (Standards for Reporting Qualitative Research) [19].

### Ethical Considerations

This study was conducted at North York General Hospital, diagnostic breast cancer and chemotherapy clinics. Informed consent was obtained from all participants across the 3 phases. All data were anonymized using study ID numbers and stored in secure locations. Data collection occurred between 2019 and 2024. Ethics approval was granted by the NYGH Research Ethics Board (protocol #18‐001 and #0326).

### Setting, Intervention, and Implementation

Our study took place at 2 ambulatory oncology settings of the NYGH (a tertiary-level, academic-affiliated community hospital in Toronto, Ontario, Canada): the BDC and the CC [20]. The BDC is a specialized ambulatory diagnostic service within the Breast Centre that provides comprehensive assessment for individuals with suspected or confirmed breast cancer, identified through abnormal clinical examination and/or breast imaging, serving approximately 1500 new patients seen annually. In contrast, the CC is an outpatient systemic therapy unit that delivers chemotherapy and supportive care to patients with confirmed cancer diagnosis (including early-stage, locally advanced, and metastatic breast cancer) coordinated by charge nurses following consultation with medical oncology, with pharmacists providing patient education and counseling. Whereas the BDC primarily serves patients in the diagnostic and decision-making phase (often pretreatment), the CC primarily serves patients in active treatment, with more advanced or definitively staged disease and correspondingly different informational, supportive, and symptom-management needs. Our implementation work, therefore, involved adapting the intervention from a diagnostic-focused setting (BDC) to a treatment-focused setting (CC), taking into account differences in patient flow, acuity (eg, treatment-related toxicities), and longitudinal relationships with the oncology team. The focus of our evaluation was the Clinical Navigation Tool within the MyJourney platform. The tool was first implemented at the BDC on September 19, 2022, as part of a hospital-wide initiative to improve cancer care delivery. It was subsequently adapted for use at the NYGH CC on March 4, 2024. The BDC manages approximately 700 patients with breast cancer per year.

### Participant Recruitment

#### Phase 1 (BDC Patients)

We used purposive sampling with convenience and self-selection elements to identify eligible participants: English-speaking adults diagnosed with breast cancer who were former or current patients receiving care at the BDC and able to provide informed consent [21]. To obtain a wide range of patient experiences, recruitment was conducted in a multipronged approach. For example, we recruited participants at the end of a 1-day workshop for newly diagnosed patients by displaying posters in the clinic waiting rooms. Second, we contacted patients who had previously indicated their willingness to participate in any future research studies. All participants spoke English and provided informed consent.

#### Phases 2 and 3 (Providers and Staff)

Across phases 2 and 3, we used purposive sampling within each clinic [21] to invite all current or prospective MyJourney users (nurses, pharmacists, and administrative staff) at the BDC and the CC, with the goal of capturing the full range of user perspectives rather than thematic saturation [22]. We did not recruit oncologists or surgeons for phase 2 because at the CC, these providers primarily interact with MyJourney through the Multidisciplinary Cancer Conference Tool and the hospital electronic medical record rather than as routine users of the CC Clinical Navigation Tool. Charge nurses and oncology pharmacists are the primary day-to-day users of the Clinical Navigation Tool, responsible for coordinating chemotherapy visits, managing treatment-related symptoms, and delivering patient education and counseling. Potential participants were invited via targeted email with support from senior clinic leaders. Written informed consent and demographic data were collected using Qualtrics. One participant did not consent to the use of direct quotes.

### Data Collection

Across phases, data collection included semistructured interviews or online surveys. The research team (IH, JM, MK, LH, and KM) developed and pilot-tested the instruments to ensure clarity, appropriateness, and alignment with the study objectives*.*

#### Phase 1

A semistructured interview guide was created and modified iteratively (Multimedia Appendix 1). In interpretive description, insights from early interviews inform the design of subsequent interviews, including the interview guide, which begins with broad, open-ended questions and evolves as themes, categories, and theoretical constructs are developed through ongoing analysis [15]. Two experienced qualitative researchers (LH and KM) conducted interviews with women who were diagnosed with breast cancer and were former or current patients, in person at the BDC. Interviews lasted 60 to 90 minutes. During the interviews, participants were asked to map out their breast cancer journey, starting from the time before their diagnosis to the present. For example, they were asked about their activities, the health professionals they interacted with, their feelings, the information they received, and their communication needs at each stage of their journey. Participants were encouraged to take the conversation in new directions and explore concepts not initiated by the interviewers. Our goal was to learn from participants’ breast cancer journeys at the BDC to identify gaps in care and their information and support needs, in anticipation of developing a patient-facing app that would address these needs as a complement to the Clinical Navigation Tool aimed at providers.

#### Phase 2

A semistructured interview guide was developed (for each of the pharmacist and nurse participants) by 3 researchers (IH, JM, and MK; Multimedia Appendix 2). Interviews were conducted in person or virtually (via Zoom [Zoom Video Communications] or Microsoft Teams) in 30-minute time blocks, at the participant’s preference. Interview guides followed a semistructured format informed by the RE-AIM framework [18] (ie, to help formulate questions that would elicit responses grounded in implementation theory). To maximize participation, we offered flexible participation options (a single 1-hour session or two 30-minute sessions). Interview questions focused on (1) participants’ roles and responsibilities, (2) experience using MyJourney (including duration and frequency of use; how it supports tasks, workflows, and patient management; and whether it meets their needs), (3) MyJourney feature-specific feedback, (4) benefits and challenges of using MyJourney and related recommendations, (5) experience with implementation (what worked well, what did not, any disruptions to workflow, and suggestions for future implementation), and (6) challenges experienced in each of these areas along with associated recommendations, including feedback on how MyJourney could be tailored to support their work better.

#### Phase 3

The research team developed an online survey for each of the BDC and CC and pilot-tested it for understandability (Multimedia Appendix 3). Surveys were tailored to different audience groups (eg, nurses and clinic staff) and administered via Qualtrics to study participants to collect data on demographics and usability. Usability and user experience were assessed using the System Usability Scale (SUS) [23,24] and the technology acceptance model, which measures ease of use and usefulness of a technology-based innovation [25,26]. Sampling was consistent with established usability guidance that 3‐5 users are typically sufficient to identify most high-severity usability problems in a given design iteration [27]. As such, we aimed to reach at least 3 participants per site who had access to and experience using the platform but were constrained by the number of eligible users during early implementation (2 patients with breast cancer per site). As a result, we treated this as an initial, depth-oriented usability assessment focused on uncovering major usability issues and gathering rich feedback to guide subsequent refinements, rather than on producing generalizable estimates of usability metrics. We also assessed perceptions of workflow alignment, ratings of specific MyJourney features, and overall satisfaction. Demographic variables included age, sex, gender, ethnocultural background, and years in role. In addition, survey responses (stage 1) were used to tailor interview probes for stage 2. For example, if a participant rated a MyJourney feature poorly, the interview explored the reasons and discussed possible solutions to address them.

### Data Analysis and Synthesis

#### Qualitative Data

All interviews were digitally recorded, transcribed verbatim, and reviewed for accuracy. For qualitative analyses across all phases, we used an iterative, inductive, and interpretive process to construct our understanding of MyJourney. Within the interpretive description methodology of Sandelowski [16], qualitative data analysis unfolds as an iterative, inductive, and reflexive process. It begins with immersion in the data to gain contextual understanding, followed by broad initial coding to capture patterns without imposing rigid categories. In phase 1, reviewers (LH and KM) used constant comparison analysis, repeatedly contrasting data segments and cases to develop, refine, and validate emerging insights and themes [16]. This process allowed us to move beyond description, producing a practice-oriented interpretation that informed actionable insights about how MyJourney should be constructed. The codebook was developed iteratively with each subsequent transcript, and its emergent structure became more focused as categories and themes developed. In phase 2, we used directed content analysis, combining deductive coding (guided by RE-AIM and study objectives) and inductive coding to capture themes emerging from participant narratives [28]. Reviewers in this phase (IH, JM, and MK) independently coded initial transcripts and collaboratively developed an initial codebook that evolved as more data were analyzed and was structured around barriers and recommendations. The remaining transcripts were coded in duplicate by applying the codebook. Discrepancies were resolved by consensus or adjudication with a third reviewer (MK). Thematic categories were refined through team discussions to ensure clarity, distinctiveness, and alignment with the study objectives. Data were then organized by clinical site and mapped onto a figure depicting the workflow, allowing the clinical context in which MyJourney would be used to be identified. Open-ended survey responses were independently coded by 2 reviewers using the same coding structure.

#### Data Synthesis

Across phases, coded qualitative data were summarized in tables organized by challenges, recommendations, and, where relevant, enabling factors. Representative quotations were selected to illustrate key findings.

#### Quantitative Data

Survey data were analyzed descriptively using Microsoft Excel. Measures included frequencies, percentages, means, medians, SDs, and IQRs. SUS scores were interpreted using the following benchmarks: >68 (above average), >70 (acceptable), >74 (excellent), and >85 (best imaginable) [29]. Mean scores and SDs were calculated for technology acceptance model dimensions [25]. Due to the small sample size, no inferential statistics were conducted.

### Researcher Characteristics and Reflexivity

The evaluation team comprised researchers who identified as female and women from health services and implementation science, who were not involved in the design or delivery of MyJourney. To mitigate potential bias arising from the dual role of a coprincipal investigator (FO) as a platform developer, data collection and analysis were conducted by team members independent of platform design and operations. The interviewer had no prior relationship with the participants. A note-taker attended phase 3 interviews and offered follow-up questions where appropriate. Team members kept reflexive journals and audit trails and met regularly to review assumptions and ground analytic decisions in participant accounts. Member checking was not feasible. Credibility was supported through rich description and direct quotation.

## Results

### Phase 1: Predesign Engagement With Patients at the Breast Diagnostic Clinic

#### Participant Characteristics

In total, 13 participants consented and were interviewed between 2018 and 2019 (Table 1). The mean age was 56 years (range 42‐67 years), all identified as female and a woman, and most identified as White (5/13, 38.5%), South Asian (2/13, 15.4%), and Chinese (2/13, 15.4%), and 1 (7.7%) participant each identified as Jewish, Latin American, West Asian, or White-Aboriginal. The majority of participants (9/13, 69.2%) reported an annual household income of ≥US $659,200. All participants spoke English, and 3 (23.1%) individuals spoke a second language. Of the 12 who specified education, most (8/12, 66.7%) reported at least a bachelor’s degree.

**Table 1.** Phase 1 participant characteristics^a^.

Characteristic	Values (n=13), n (%)
Age range (n=10)	
41‐50 years	2 (20)
51‐60 years	4 (40)
>60 years	4 (40)
Prefer not to say	3 (23.1)
Biological sex	
Female	13 (100)
Gender identity	
Woman	13 (100)
Ethnic or racial background	
Chinese	2 (15.4)
Jewish	1 (7.7)
Latin American	1 (7.7)
South Asian	2 (15.4)
West Asian	1 (7.7)
White	5 (38.5)
White-Aboriginal	1 (7.7)
Education (n=12)	
University—bachelor or more	8 (66.7)
College diploma	3 (25)
University—no degree	1 (8.3)
Prefer not to answer	1 (7.7)
Languages spoken^b^	
English	13 (100)
Arabic	1 (7.7)
Russian	1 (7.7)
Chinese	1 (7.7)
Household income (n=10)	
US $110,000	4 (40)
US $66,000-$88,000	3 (30)
US $88,000-$110,000	2 (20)
US $0-$22,000	1 (10)
US $22,000-$66,000	0 (0)
Prefer not to answer	3 (23.1)
Stage of breast cancer journey (n=13)	
Prediagnosis	2 (15.4)
Diagnosis	3 (23.1)
Treatment	4 (30.8)
Survivorship	4 (30.8)

a Percentages may not add to 100% due to rounding.

b The totals in this section of the table are greater than the sample size, and the percentages add to more than 100%.

#### Stage of Breast Cancer Journey

**Figure 2.**
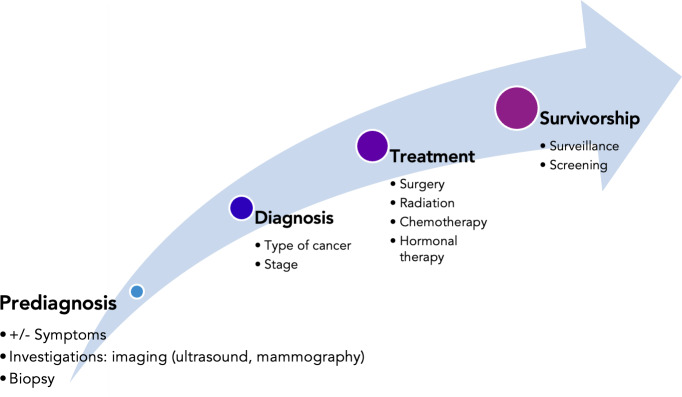
Stages of the typical breast cancer journey as reported by interview participants.

Participants described different “phases” of their journey, each with its own associated challenges and communication needs (Figure 2). The phases identified were “prediagnosis,” “diagnosis,” “treatment” (including active management strategies such as surgery, chemotherapy, and radiation), and “survivorship.” These labels mark the key clinical activities during the respective time frame and reflect the clinical perspective that patients with breast cancer are expected to adopt. The majority of participants were at the treatment (4/13, 30.8%) or survivorship (4/13, 30.8%) stage, followed by the diagnosis (3/13, 23.1%) and prediagnosis (2/13, 15.4%) stages. However, participants acknowledged that these phases were not always linear, and not all patients go through all phases. Participants reported that each phase had distinct informational and emotional needs, which were incorporated into the tool’s design features. Multimedia Appendix 4 summarizes the themes and relevant quotes describing participants’ experiences according to their breast cancer journey stages.

#### Summary of Qualitative Results

##### Overview

We developed 8 overarching themes that describe participants’ experiences across the breast cancer care trajectory at the BDC and CC at NYGH (Table 2).

**Table 2.** Overarching themes that describe participants’ experiences across the breast cancer care trajectory at the North York General Hospital Breast Diagnostic Centre (BDC) and Chemotherapy Clinics.

Theme and subtheme	Description	Illustrative quotes
1. Care coordination and workflows		
BDC workflows and processes	Perceptions of the BDC as coordinated, efficient, and well-organized.	“From the time from mammogram to surgery was only 1 month—that was an amazing experience” [Participant 7]. “They have a very good process ... After your appointment [they] give you another one ... If you need a referral, they have already sent the referral ... It’s a very good process and I don’t want it to change” [Participant 1].
Transitions and follow-up	Breakdowns when moving between BDC, chemo, radiation, home care, and primary care, with patients worrying that things will be missed.	“At the BDC it was a real team approach ... Then, when I went to chemo there was no team approach and it all went downhill from there” [Participant 4]. “The imaging follow up just doesn’t happen. I have to orchestrate it” [Participant 4].
2. Communication and information flow		
Receiving diagnosis	How, where, and by whom the diagnosis is communicated, and its emotional impact.	“When I got the phone call from my doctor, I was alone at home and told about the diagnosis. That was hard. It would have been better to have someone with me” [Participant 7]. “I was ‘told’ that I had breast cancer by the bone scan technician ... before I was given the official diagnosis and that was difficult to hear and very brusque” [Participant 8].
Team communication and contradictions	Inconsistent or conflicting information between clinicians and services, eroding trust.	“There was lack of communication between groups in this hospital ... I spent the whole week panicking ... and they said ‘oh it’s fine, we have ice for you’” [Participant 4]. “They are telling you it’s nothing ... They are not doing their research to understand the actual side effects of radiation ... Every time you say it, they dismiss it instead of cataloguing people’s experience” [Participant 18].
Role clarity (who to call)	Uncertainty about which clinician is responsible for which problems.	“There were a few things that I wasn’t sure if I should go to the surgeon or the medical oncologist for ... I often wasn’t sure who to go to” [Participant 9].
Tone, empathy, and respect	Value placed on clear, respectful, and nondismissive communication.	“We had an amazing surgeon and oncologist ... They don’t try to make you feel bad ... They never show signs of exasperation ... It’s very personalized” [Participant 1].
3. Information and resource needs		
Volume and timing of information	Tension between information overload and unmet information needs; importance of timing.	“They gave me lots of information in the little bag. It was overwhelming ... When I started reading it, it was okay” [Participant 5]. “The ‘welcome to breast cancer’ package ... had too much of a lumpectomy focus versus mastectomy” [Participant 4].
Practical “how-to” guidance	Desire for concrete, practical advice about surgery recovery, daily logistics, and self-care.	“I thought I’d get more info ... what to expect and prepare for after the surgery ... mastectomy bras, etc. I did research online and got a wedgie pillow ... grabbers ... special tables ...” [Participant 2].
Understanding diagnosis and treatment	Need to understand pathology, staging, oncotype, systemic therapies, and their implications.	“I was trying to understand the HER2. I found this confusing ... It would be good to ... tell us which sites to go to, and not just Google” [Participant 9].
Survivorship “what happens after”	Lack of clear survivorship plans, follow-up schedules, and guidance for life after treatment.	“After the treatments were over, I was left alone. I want to know, what happens after cancer? ... Now that I have had cancer, it has changed me. How do I deal with this?” [Participant 9].
Safe, reputable online information	Desire for trusted, curated web resources instead of unguided internet searches.	“I was googling a lot—wish I’d had something reputable to refer to” [Participant 15].
4. Digital tools and patient portal		
Access to records and results	Strong interest in a portal centralizing reports, laboratory results, and imaging.	“I would like a patient portal with all of my reports and bloodwork results ... upcoming appointments, information on drugs I’m taking, and resources, like Wellspring” [Participant 6]. “She loves Sunnybrook mychart ... she can bring the results to other medical appointments, so they have the information” [Participant 4].
Appointments and chronology	Desire for a clear visual timeline of appointments and treatments.	“Would like to see the chronology of her appointments and treatments in a” [Participant 7].
Laboratory result “lookup”	Need for plain-language explanations of test results.	“What about a lookup feature for what the lab results all mean—this would be great, so you don’t have to go Google” [Participant 8].
Messaging and chat	Preference for asynchronous written communication for nonurgent questions.	“A good option would be to have a chat function ... I don’t necessarily need an answer right away and I don’t want to talk on the phone” [Participant 3].
Technology comfort and training	Wide variation in digital literacy and format preference (portal vs paper).	“I would be very interested in using the portal, but someone would have to show me how to use it because I’m not good at using computers” [Participant 5]. “I am a paper person. Would like to have all the educational materials paper-based” [Participant 8].
5. Experience of quality of care		
BDC environment and team	Perceptions of the clinic environment, staff attitudes, and teamwork.	“The whole team—everybody. Every time I am here I feel that I am coming home” [Participant 1].
Chemo and radiation experiences	Physical and emotional experiences of systemic and radiation treatments.	“Chemo is the worst thing ever ... The chemotherapy treatments involved long waits” [Participant 6]. “The nurses there were amazing at the chemo clinic ... They really tried to make a really bad situation bearable” [Participant 4].
Home care or CCAC^a^	Challenges with community nursing, reliability, and competence.	“CCAC came and took care of my wound. It was a comedy of errors ... They had no idea whether they were coming or going, and whether they had supplies” [Participant 4].
6. Psychosocial and emotional support		
Distress and uncertainty	Intense distress at diagnosis and during periods of waiting or uncertainty.	“The hardest part was from when I thought I probably had breast cancer, but didn’t know for sure ... The hardest part was not knowing whether I would be able to see my son go to school” [Participant 16].
Ongoing anxiety and fear of recurrence	Persistent psychological impact after treatment ends.	“I still struggle with the psychological aftercare ... You are always on guard for another lump” [Participant 9].
Support services and gaps	Value of programs like Wellspring and perceived gaps in psychosocial support.	“The hospital just focuses on the body. They don’t focus on the mental and the cognitive. I was happy to find Wellspring ... I go to a lot of classes” [Participant 6].
Humor and coping styles	Use of humor and individualized coping strategies.	“Am I stressed, absolutely. To deal with it, I sit in the car and do stand-up humour for myself” [Participant 5].
7. Patient agency and self-management		
Self-advocacy and orchestrating care	Patients pushing for referrals, chasing results, and coordinating their own care.	“I have to orchestrate it ... If you aren’t willing to drive the car, let me. I want my test results. I want them to be accessible” [Participant 4].
Personal tracking and journaling	Using notebooks, binders, and journals to track care and questions.	“After each appointment, I wrote in my book what happened. So, I have this record of what care I received” [Participant 12].
Doing own research and lifestyle change	Independent research on treatments and lifestyle changes to support recovery.	“I wanted to be part of my recovery, so I did some digging and reading ... I removed sugar from my diet ... removed meat ... took 2 minute cold showers ... and drank a lot of water before chemo” [Participant 3].
8. Survivorship care and transitions		
Who leads survivorship	Confusion and preferences around oncologist- versus GP^b^-led follow-up.	“What happens now? Who follows me? Who do I speak with? ... There is no coordinated care. No one is in charge. It’s you” [Participant 18]. “I would prefer having my GP follow me once my treatment is complete ... She phones me and tells me my results” [Participant 11].
Requirements for GP-led care	Perceived capabilities and training needed in primary care.	“The family doctor would have to have a lot of training regarding ... post-treatment side effects ... and the softer skills side of it” [Participant 7].
Long-term anxiety and follow-up	Ongoing fear of recurrence and desire for continued monitoring as years pass.	“As the years go by, you think, nothing has happened. And you start to worry ... That’s when the anxiety starts to come back with the waiting” [Participant 13].
Survivorship identity and peer support	Seeking peer support that matches age, life stage, and experience.	“I would be really interested in a survivorship focus group ... After the treatments were over, I was left alone” [Participant 9]. “I still haven’t found someone like me ... a mother of two young kids ... To have that kind of peer support is really powerful” [Participant 16].

a CCAC: community care access center.

b GP: general practitioner.

##### Care Coordination and Workflows

Participants described the BDC as a coordinated, efficient entry point into care, characterized by rapid movement from abnormal imaging to biopsy and surgery and by clear internal communication. Women emphasized short wait times and proactive scheduling of subsequent appointments and referrals, which reduced uncertainty and signaled that “the system” was functioning well. In contrast, this perceived coordination often deteriorated once patients transitioned into chemotherapy, radiation, community nursing, or primary care. Several participants reported that the “team approach” appeared to end after surgery, with no single clinician or service clearly overseeing the remainder of care. Delays in imaging follow-up, confusion around orders, and inconsistent tracking of test results were frequently described. As a result, many participants felt responsible for navigating and organizing their own care, including obtaining their results, confirming appointments, and ensuring referrals had been made, a role they found exhausting in the context of illness and emotional distress.

##### Communication and Information Flow

How and where the diagnosis was communicated strongly shaped participants’ early experiences. Some received the diagnosis alone at home by telephone and described this as particularly distressing; others were informed indirectly by nonphysician staff (eg, imaging technicians) before any formal consultation, which they found abrupt and insensitive. Beyond diagnosis, participants highlighted inconsistent messages between clinicians and services and poor intrateam communication, including contradictory advice between oncology and treatment units, eroding trust and increasing anxiety. Many were uncertain whether to contact their surgeon, medical oncologist, radiation oncologist, family physician, or nurse navigator when new questions or symptoms arose. At the same time, participants expressed high appreciation for clinicians who communicated in clear, plain language, invited questions, and repeated information without frustration. Participants also reported that initial information packages were sometimes overwhelming and poorly tailored (eg, lumpectomy-focused materials given to women undergoing mastectomy) and found gaps in practical “how-to” guidance (eg, what to buy and prepare before surgery, managing drains, and concrete strategies for side effects) and in survivorship-oriented information about “what happens after” treatment, follow-up schedules, and recurrence risk.

##### Information and Resource Needs

Information needs were complex and shifted across the cancer trajectory. Many participants recalled receiving large information packages at diagnosis that felt overwhelming or poorly tailored (eg, materials focused on lumpectomy when they were having mastectomy), even as they still lacked answers to very practical questions about what to expect after surgery and how to prepare their home. They sought clear, accessible explanations of pathology results, risk scores, and treatments, as well as guidance on what is “normal” versus concerning during and after treatment. Critically, once active treatment ended, people described a stark information void about survivorship—wanting to know what happens next, how long follow-up would last, what symptoms to watch for, and how to live with the ongoing impact of cancer. Many expressed a desire for curated, trustworthy online resources to reduce reliance on unsupervised internet searches.

##### Digital Tools and Patient Portal

Participants suggested that a cancer-focused portal to address multiple unmet needs in one place would be helpful. Those with experience of existing portals valued having access to laboratory results, imaging reports, and appointment lists, and being able to bring printed results to other appointments. They imagined a more tailored tool that would provide a clear chronology of appointments and treatments, explanations of laboratory values in plain language, and secure messaging or chat for nonurgent questions. However, our data also highlighted wide variation in participants’ comfort level with digital solutions: some described themselves as “information junkies” who would “be on it all the time,” while others said they would need to be walked through how to use such a system or preferred paper-based materials. Any digital solution would therefore need to support both high- and low-technology users, offer training, and coexist with paper formats rather than fully replace them. Multimedia Appendix 5 summarizes the features participants reported they would be interested in seeing in a future patient portal.

##### Experience of Quality of Care

The overall quality of care was experienced as uneven. Many participants spoke positively about the BDC environment, highlighting friendly staff, clean, welcoming spaces, and a sense of being “at home” and well-supported. Chemotherapy units were described as both physically and emotionally challenging, yet often made more bearable by attentive, kind nurses. In contrast, community-based home care services were frequently characterized as disorganized, inconsistent, or poorly coordinated, with missed visits, questionable technique, or lack of supplies. Long waits for some specialist appointments, crowded spaces, and privacy concerns in certain treatment areas also shaped perceptions of quality. These contrasting experiences underscored how much the attitudes, organization, and resources of specific teams influence patients’ sense of safety and trust.

##### Psychosocial and Emotional Support

Across the cancer journey, participants reported substantial psychosocial burden. The period between suspecting cancer and receiving a definitive diagnosis was described as especially distressing, particularly for those with young children who feared not living to see key milestones. They described intense fear and uncertainty at the time of diagnosis and during periods of waiting for test results or treatment decisions. Even after treatment, many reported ongoing psychological impacts, including heightened worry before follow-up imaging and a sense of being “on guard” for recurrence. Some participants found that strong support through community-based survivorship centers (such as psychosocial programs and survivorship classes) was essential to addressing emotional and cognitive impacts that hospital-based services, which “just focus on the body,” often neglect and do not adequately address mental health, cognitive changes, or other concerns. Participants emphasized the value of humor and individualized coping strategies, and having space for levity, storytelling, and emotional validation alongside more formal counseling or group support. Transitions into survivorship were characterized by anxiety and uncertainty about who was responsible for ongoing care. Some participants were comfortable transitioning to a trusted family physician, while others questioned whether primary care had sufficient oncology expertise and time to manage late effects and psychological needs. Many described feeling “left alone” once active treatment concluded.

##### Patient Agency and Self-Management

Across interviews, patient participants portrayed themselves as active agents who often had to fill gaps in the system. Many described pushing for referrals, insisting on further tests, or repeatedly calling to obtain results, reflecting both a strong desire for control and a lack of trust that the system would automatically “catch” everything. Self-management practices were common. For example, participants kept notebooks or binders to track appointments, results, and questions, and some engaged in extensive independent research on treatments, nutrition, exercise, and complementary strategies to support recovery. These behaviors were framed both as empowering and burdensome, as patients felt they “shouldn’t have to” organize their own care to this degree; yet, they would have valued tools and information that would have allowed them to do these activities more effectively.

##### Survivorship Care and Transitions

The transition from active treatment to survivorship was a major source of uncertainty and distress. Participants frequently reported not knowing who was now “in charge” of their care, how often they would be seen, or what tests would be done in follow-up. Some were comfortable with their family physician assuming a lead role, particularly when they had longstanding, trusting relationships and good access. However, others doubted that primary care providers had sufficient oncology-specific knowledge or time to manage their physical and psychological needs without additional training. Survivorship was also described as a distinct identity shift, whereby they no longer felt like active patients but did not feel “back to normal” either. Many sought survivorship programs, focus groups, or peer connections that recognized and supported this ongoing phase, rather than treating the end of treatment as the end of the cancer experience.

### How Findings Informed the Design of the Patient-Facing App of MyJourney

Overall, participants emphasized the importance of structured follow-up plans, clear communication about recurrence risk, and accessible survivorship resources. Table 3 highlights the features identified by participants as to be considered for the patient-facing app. Multimedia Appendix 6 presents the minimum viable feature set used to build the patient-facing app.

**Table 3.** Features that were considered in the development of the patient-facing app.

App feature area	Directly addresses participant needs or quotes
Timeline and roadmap	Need for chronology, pathway or flowchart, and “knowing what happens next” including aftercare and survivorship.
Results and lookup	Desire for laboratory and test access, explanations (oncotype, seed lock, and radiation effects), and faster and clearer results.
Messaging and routing	Confusion about who to contact, wish for chat, frustration with missed callbacks, and vacation gaps.
Education library	Information overload plus gaps; wish for curated sites, practical tips, emotional support, and survivorship information.
Psychosocial or peer support	Requests for psychologists, survivorship groups, younger-women groups, peer stories, and contact.
Self-management tools	Patients documenting care, tracking steps, changing diet, and orchestrating follow-up themselves.

### Phase 2: Preimplementation Interviews at the CC

#### Participant Characteristics

In total, 3 nurses and 5 pharmacists were interviewed between November 23 and 29, 2023, representing all relevant users of the MyJourney platform at the CC. Most participants (7/8, 87.5%) identified as female and a woman, aged 41‐50 years (5/8, 62.5%), primarily with a Chinese (2/8, 25%), East African (1/8, 12.5%), South Asian (3/8, 37.5%), or White (2/8, 25%) racial background. Most participants had been in their current role for under 5 years (5/8, 62.5%; Table 4). At the CC, nurses were primarily responsible for coordinating care, managing symptoms, and answering patient inquiries. Pharmacists provided patient education, addressed treatment-related issues, secured funding, and offered tailored resources. Two pharmacist roles existed: Clinical One, who conducted scheduled education sessions before the first chemotherapy cycle, and Clinical Two, who provided chairside counseling to patients in clinical trials or to those who had not received prior education on the day of their first treatment.

**Table 4.** Phase 2 participant characteristics^a^.

Characteristic	Values (n=8), n (%)
Age range (years)	
30‐40	1 (12.5)
41‐50	5 (62.5)
51‐60	2 (25)
>60	0 (0)
Biological sex	
Female	7 (87.5)
Male	1 (12.5)
Other	0 (0)
Gender identity	
Woman	7 (87.5)
Man	1 (12.5)
Other	0 (0)
Ethnic or racial background^b^	
White	2 (25)
Chinese	2 (25)
Black	0 (0)
Chinese	2 (25)
East African	1 (12.5)
Latin American	0 (0)
South Asian	3 (37.5)
White	2 (25)
East African	1 (12.5)
Prefer not to say	2 (25)
Years in current role	
<3	1 (12.5)
3‐5	4 (50)
6‐10	1 (12.5)
>10	2 (25)

a Percentages may not add to 100% due to rounding.

b The totals in this section of the table are greater than the sample size and the percentages add to greater than 100%.

#### Workflow Mapping: Phases of Chemotherapy Care

**Figure 3.**
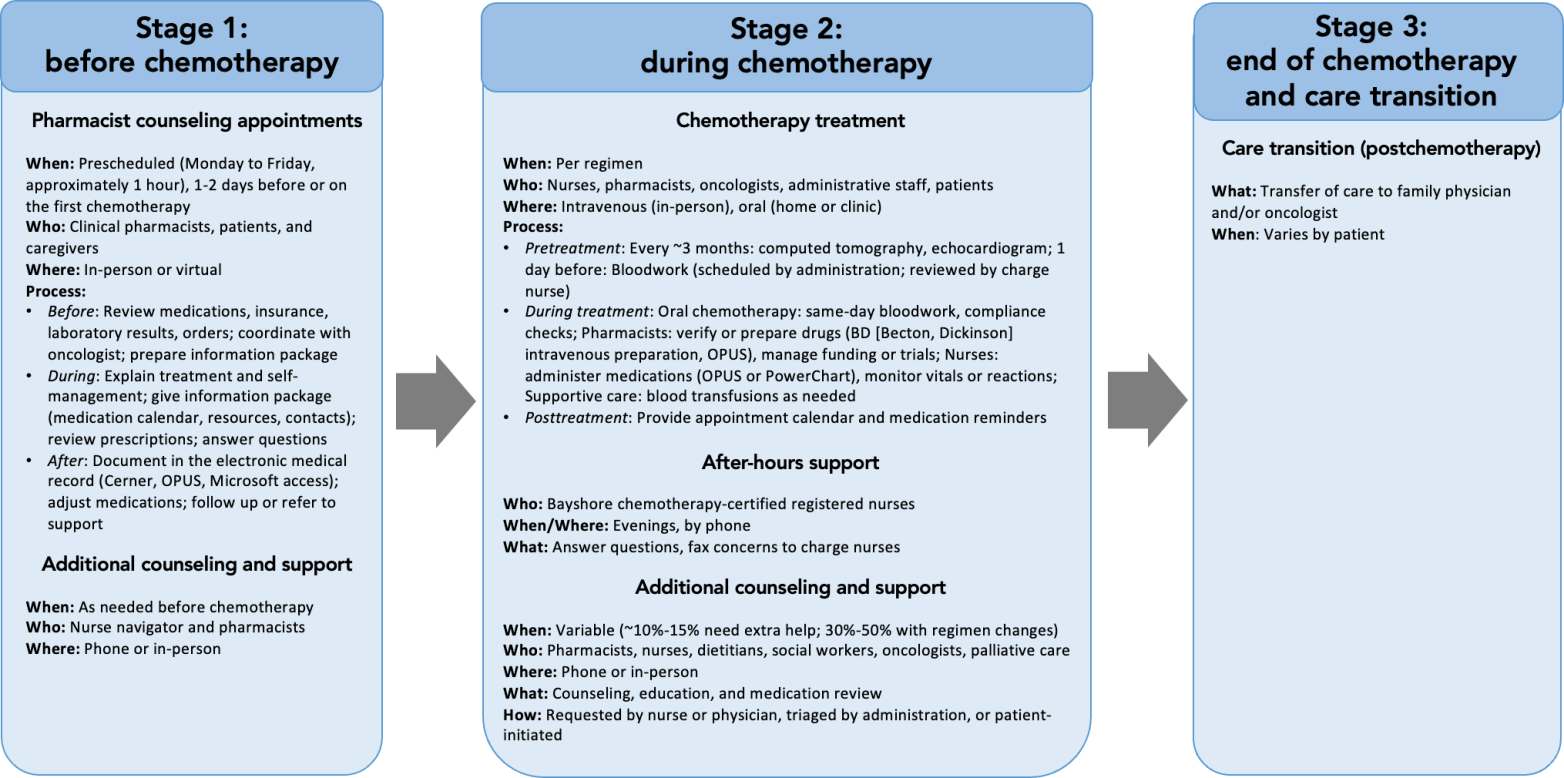
Workflow stages during chemotherapy treatment. OPUS: Ontario Public Health Standards.

Workflows described by participants spanned three stages of the patient journey during their chemotherapy treatment: (1) before chemotherapy begins (including pretreatment education and preparation), (2) during chemotherapy (including treatment delivery, monitoring, and follow-up), and (3) end-of-treatment and transition to the next phase of care (Figure 3).

#### Challenges and Recommendations

Preimplementation interviews with pharmacists and nurses uncovered multilevel challenges to care coordination across the chemotherapy care continuum, which we organized into five domains: (1) patient-level, (2) administrative and workflow inefficiencies, (3) health information technology systems limitations (eg, poor electronic health record usability and lack of interoperability between Ontario Public Health Standards, Cerner, and Connecting Ontario), (4) health resource constraints (eg, nurse staffing shortages and inadequate physical space), and (5) quality of care gaps (eg, lack of pharmacist continuity and no secure patient communication channels). Table 5 summarizes these challenges and MyJourney design recommendations to address them across the 3 stages of chemotherapy care: stage 1 (before), stage 2 (during), and stage 3 (end).

**Table 5.** Implementation challenges across the chemotherapy care continuum and MyJourney design recommendations to address them.

Challenge domain	Stage 1: before chemotherapy (Multimedia Appendix 7)	Stage 2: during chemotherapy (Multimedia Appendix 8)	Stage 3: end of chemotherapy and transition (Multimedia Appendix 9)	MyJourney design recommendations to address challenges
1. Patient-level	Lack of access to appointment information; privacy concerns about digital resources; limited digital literacy and technology access.	Information overload when large volumes of information were delivered on treatment days; walk-in patients seeking urgent support; limited after-hours access to clinicians.	Regional differences in community care coordination contributing to incomplete medication information and gaps at transition.	Patient-facing app centralizing appointment details and visit summaries; layered, on-demand education to reduce overload; mobile-friendly, plain-language content to support varying digital literacy; future secure messaging to support questions between visits and during transitions.
2. Administrative and workflow	Incomplete or unsigned prescriptions; late counseling notifications; delayed funding reviews; manual preparation of regimen-specific materials; heavy follow-up demands.	Reliance on verbal updates or email for after-hours issues; incomplete information leading to duplicated counseling; nurses unaware of appointment changes; high call volumes; manual record checks and after-hours reports.	Regional community care access differences hindering coordination and resulting in incomplete medication lists at transition.	Clinical Navigation Tool supporting task lists, standardized documentation templates, and structured prompts; centralized record of counseling and follow-up to reduce duplication; integration of key appointment and treatment information to support proactive planning and smoother transitions.
3. Health IT systems	Time-intensive Cerner documentation; restricted access and incomplete patient information; limited interoperability between OPUS^a^, Connecting Ontario, and PowerChart; pharmacists unable to view oncologist appointments.	Manual order entry and frequent program switching; lost interprofessional notes on admission; unclear note headings; ongoing limited interoperability between OPUS and Cerner.	Fragmented information across hospital and community systems contributing to incomplete medication histories.	MyJourney designed as a single navigation workspace that aggregates key information from multiple systems where possible, reduces duplicate data entry through structured fields, and surfaces oncology appointments and treatment plans in a consolidated view to support navigation across settings.
4. Health resources	Complex funding confirmation processes; strained patient-to-nurse ratios; staffing constraints; shared counseling space with a vaccine clinic.	Nurse staffing shortages; physical space limitations restricting in-clinic transfusions; high-risk treatments requiring extended monitoring.	Ongoing resource constraints in community and hospital settings affecting follow-up capacity.	Workflow tools to streamline preparation for counseling, reduce repetitive administrative tasks, and support team-based navigation so workload can be shared across pharmacists and nurses, with clearer task allocation over time.
5. Quality of care	Medication charts lacking visuals or digital access; strict time limits that truncate counseling; privacy concerns limiting use of educational videos; inefficient coordination of bloodwork and education appointments.	Delayed drug preparation prolonging chair time; no 24/7 clinician availability; no dedicated pharmacist continuity; no secure electronic communication with patients.	Coordination gaps at transition leading to fragmented follow-up and incomplete information for community providers.	Visual, phase-specific patient education content; structured symptom and concern tracking to focus limited visit time; clear assignment of navigation tasks within the care team to enhance continuity and follow-up; digital information sharing to support more consistent transitions.

a OPUS: Ontario Public Health Standards.

Briefly, across stages, staff described patients’ difficulty accessing technology, tracking appointments, and experiencing information overload when large volumes of education were delivered on treatment days and had limited options for secure communication and follow-up between visits. They also emphasized administrative inefficiencies (eg, incomplete prescriptions, delayed notifications, and duplicated counseling); manual, fragmented, and time-intensive documentation across Cerner, Ontario Public Health Standards, and Connecting Ontario; staffing and space constraints; and coordination gaps at transitions to community care. These findings contributed to the design of MyJourney’s patient-facing app (centralized appointment and treatment information, phase-specific educational content, and tools to support symptom tracking) and the Clinical Navigation Tool (structured prompts, standardized documentation templates, and a shared workspace to reduce duplication and improve visibility across the team), with the goal of addressing the specific workflow, communication, and information-access barriers identified by frontline clinicians and staff. See the complete set of challenges, recommendations, and related participant quotes for each stage in Multimedia Appendices 7-9.

### Phase 3: Evaluation of MyJourney’s Implementation and Usability

#### Participant Characteristics

In total, 2 charge nurses (CC), 1 charge nurse, and 1 administrative staff member (BDC clinic) participated in the usability evaluation. This sample comprised female participants aged 30‐60 years who identified as women, with Black, East African, South Asian, and White racial backgrounds. Participants had been in their professional roles for 5‐11 years (Multimedia Appendix 10). Phase 3 findings primarily reflect the RE-AIM domains of implementation and maintenance. Implementation-related results (stages 1 and 2) describe how patients navigated the app, the extent to which MyJourney fit within their routines, and specific usability issues that affected completion of key tasks, whereas maintenance-related results (stage 3) focus on participants’ intentions to continue using MyJourney and their views on its ongoing role in their cancer journey

#### Stage 1 Evaluation (Usability Survey Results)

##### BDC Clinic

Usability was rated as excellent (mean SUS score 81.3, SD 8.8). Participants agreed that MyJourney was useful (mean score 3.9 of 5) and easy to use (mean score 4.0) and were very satisfied with the platform overall (mean score 4.5). Staff described MyJourney as helpful for workflow and daily tasks, with appointment visibility and patient organization consistently rated as easy to use, well-organized, functional, and satisfying. Notifications and diagnostic information were also valued, although perceptions of helpfulness varied slightly by role: “For me, it’s about having fewer places to look ... it helps me do my job better and save time” (Participant 09, BDC).

##### Chemotherapy Clinic

Usability was rated as excellent with a mean SUS score of 76.3 (SD 8.8)6. Participants consistently found the platform useful (mean score 3.9 of 5), very easy to use (mean score 4.3), and very satisfied overall (mean score 4.5). Features were considered understandable, well-organized, functional, and helpful, with satisfaction ranging from agree for notifications and task reminders to strongly agree for treatment summaries, appointment views, and notetaking: “It’s very easy to use and organized ... instead of clicking or going to a million places in Cerner, it’s right there” (Participant 01, CC).

### Stage 2 Evaluation (Interview Results)

#### BDC Clinic

Interviews supported survey results. Participants described MyJourney as efficient, with clear benefits for referrals, patient tracking, and information management. Shared visibility across the care team was considered a strength, and staff emphasized the value of appointment visibility, triage functions, and diagnostic notifications to support workflow. Participants also identified challenges, including glitches such as missing or outdated appointments, inconsistent notifications, and the absence of document upload capability. Recommendations included adding accountability features, digitizing paper charts, allowing customizable lists, and eventually integrating patients into multidisciplinary cancer conferences. Despite these challenges, staff expressed strong support for the platform. They described MyJourney as a communication tool that saves time, prevents patients from being missed, and offers a clear improvement over older workflows. Long-term use was considered both feasible and beneficial, with participants anticipating continued improvements and the forthcoming patient app (see Multimedia Appendix 4 for representative quotes).

#### Chemotherapy Clinic

Interviews reinforced survey findings, with participants consistently describing MyJourney as easy to use, clearly organized, and helpful for daily workflow. Staff highlighted that it took only minutes to learn to navigate the system, which replaced paper-based tracking and consolidated treatment information into a single platform. Features such as notes, tasks, treatment summaries, and appointment views were identified as particularly valuable. At the same time, participants reported challenges with reminders and notifications that were only visible during active sessions. They recommended adding email notifications and requested further integration of pathology, bloodwork, and imaging results. Limited customization and the timing of installation during work hours were also reported as concerns. Participants recommended greater flexibility in tailoring views, broader integration with existing systems, and extending MyJourney beyond breast cancer to other types of cancer.

### Stage 3 Evaluation (Repeat Usability Survey at the CC)

At the 6-week follow-up, perceived usability increased from a mean SUS score of 76.3 (SD 8.8) to 86.3 (SD 1.8). This score is interpreted as “best imaginable usability” [24]. Participants found the platform useful (mean score 4.0 of 5) and very easy to use (mean score 4.8 of 5), and they were overall very satisfied (mean score 4.5). Overall, participants expressed strong support for MyJourney, reporting that it streamlined workflows by addressing challenges identified in phase 2, showed perceived improvement in communication within the care team, and was feasible for long-term use. Perceived benefits were described as: “... everything is right there and ... you’re able to see a snapshot” (Participant 01, CC); “Before I used to have to check Cerner and SharePoint ... Now a lot of times I can just look in one place in MyJourney” (Participant 09, BDC). Based on our collective findings, five MyJourney features were developed to meet the needs of staff at the BDC: (1) diagnostic imaging and pathology notifications, (2) upcoming appointment information, (3) triaging patients accordingly, (4) diagnosis information for patients with cancer, and (5) organizing BDC patients according to their appointment types. At the CC, six features of MyJourney were identified to address the identified challenges: (1) treatment summary; (2) upcoming or past appointments view; (3) customized notes, tasks, and reminders; (4) task reminders at the patient level; (5) educational content via mobile app; and (6) overview of bloodwork appointments or results. Multimedia Appendix 5 summarizes recommendations to address challenges for specific MyJourney features.

### Technical Integration of MyJourney

Collectively, findings from each phase were translated into phase-specific prompts, content scheduling, and task workflows for both the Clinical Navigation Tool and the patient app. Regarding technical integration, the only data element entered directly by patients was the provincial health card number. All other core patient demographics (name, gender, date of birth, and age) were retrieved automatically from the NYGH electronic medical record system (Cerner) via system integration. For each patient, we linked the hospital medical record number and health card number to an existing referral in our system. If no referral record was present, we created one and manually entered the patient’s demographic information. In practice, referrals were available for the vast majority of patients, as clinic policy requires a referral from a family physician prior to consultation. Cancer diagnoses were recorded manually within the innovation platform using a structured drop-down field. Following completion of the diagnostic work-up, the confirmed diagnosis was entered to transition the patient into the “cancer diagnosis” pathway, which, in turn, enabled automated capture of subsequent hospital visits and oncology appointments. For patients with stage IV disease, clinicians manually advanced the record to the stage IV journey by selecting a dedicated “stage 4” option after the diagnosis was entered; this step was based on clinical staging, including cross-sectional imaging demonstrating distant metastases. All remaining data were captured automatically through integration with the electronic medical record. The system continuously identified relevant laboratory results and scheduled appointments, moved patients into the “cancer journey” based on appointment data, updated their status across treatment states, and generated treatment summaries from encounters with chemotherapy, surgery, and radiation without additional manual data entry.

## Discussion

### Principal Findings

Findings across the 3 phases of this research demonstrate how patient and provider input shaped MyJourney, how it functioned in practice to support the breast cancer journey for providers and patients, and its potential to reduce administrative burden while improving care coordination. Our multiphase studies demonstrated that MyJourney was feasible to implement in 2 oncology settings at an academic community hospital in Ontario, Canada, and that end users found it highly usable and helpful. The phased, iterative approach to our methods enabled insights from patients and providers to inform the design, local adaptation, and early evaluation of MyJourney, which likely contributed to its rapid acceptance.

Phase 1 provided a patient-centered map of the breast cancer journey, along with key features to be incorporated into the design of the MyJourney platform components. This phase provided foundational input for both design and implementation. Patients described the BDC as a coordinated entry point into breast cancer care but highlighted substantial gaps when transitioning into chemotherapy and survivorship, including information overload, unclear points of contact, and a need to self-manage coordination across services. For example, these insights identified the timing and content of the navigation touchpoints for the Clinical Navigation Tool and the patient app. Although this paper primarily focused on the provider-facing tool (ie, clinical navigation), the phase 1 findings are foundational, demonstrating how patient priorities can be systematically translated into platform design.

Phase 2 revealed workflow pain points in information access, communication, task tracking, and system interoperability at the CC. Nurses and pharmacists described heavy reliance on manual processes, incomplete or inconsistent appointment information, and fragmented systems. These findings positioned MyJourney as a practical solution by consolidating information, reducing manual entry, and providing notes, tasks, and reminders that supported cognitive load. The preimplementation assessment generated site-specific requirements that informed the configuration prior to rollout. These insights were used to define navigation touchpoints, content, and features in MyJourney, particularly around transitions from diagnosis to treatment and beyond, and were subsequently leveraged, alongside CC workflow data from phase 2, to guide the localization of MyJourney for the CC. By systematically mapping observed challenges to specific MyJourney design recommendations (Table 5), we demonstrated how frontline implementation barriers informed the platform’s core functions, rather than designing in isolation from workflow realities. This alignment between identified needs and design decisions is likely to have contributed to early acceptability and perceived implementation fit.

Phase 3 findings indicated strong acceptance among tool users, with improvements in usability over time. Early acceptance after only 1.5 weeks likely reflects the value of preimplementation work and the platform’s maturation since its initial BDC deployment in 2022. Staff described mechanisms through which the platform delivers benefits: replacing paper-based notes and task tracking, centralizing essential patient information, reducing duplicate system checks, and externalizing memory work through notifications and reminders. Treatment summaries and appointment views created a shared mental model across team members, which supported coordination and handoffs. Together, the layout, content, and features of MyJourney coalesced to save time and improve efficiency for daily workflows and tasks. RE-AIM also helped us to interpret phase 3 findings, revealing that, despite the small usability sample, MyJourney demonstrated promising implementation, with participants able to complete core tasks and articulating clear benefits for organizing information and tracking care. At the same time, their reflections on when and how they would continue to use the app, and on conditions needed for ongoing integration into clinical workflows, directly informed our understanding of maintenance and our plans for scale-up and refinement.

Opportunities for improvement were clear and actionable. Reliability needed to be strengthened by resolving errors to maintain user confidence, particularly during busy periods. For example, appointment views need to display entries consistently. Integrations should be expanded to include bloodwork, diagnostic imaging, and pathology, with straightforward navigation to source systems where embedding is not yet possible. Additional patient details, the ability to upload key letters or documents, and role-based list filtering would better align the tool with daily work. Out-of-app notifications, such as email triggers for reminders, could support time-sensitive follow-up when users are not actively in the system.

### Implications for Practice and Policy

The results confirm and extend existing evidence regarding the benefits and challenges of digital health platforms in oncology care. Oncology nurse navigation programs are well established and have been shown to reduce barriers, improve timely care, and enhance patient experience, particularly for patients with complex cancer trajectories [30-32]. However, navigation activities are often supported by fragmented tools (eg, electronic health record notes, spreadsheets, and paper trackers) with limited interoperability or standardized digital support for scheduling, task management, and program-level data use [31,33]. Our findings suggest that MyJourney extends these traditional models by embedding navigation functions within an integrated, provider-facing platform that consolidates appointments, treatment summaries, key laboratory information, and navigation tasks for charge nurses, pharmacists, and administrative staff in the CC. In contrast to models that rely on a single nurse navigator to manually orchestrate care, MyJourney distributes navigation activities across roles through shared dashboards and reminders, while automatically drawing core patient and appointment data from the electronic medical record to reduce duplicate documentation. By localizing the Clinical Navigation Tool to the CC, this study demonstrates how navigation principles can be operationalized within the day-to-day delivery of systemic therapy, complementing but not replacing existing nurse navigation. It also addresses calls for interoperable, scalable digital infrastructure to support navigation across the cancer continuum, which is largely lacking [30,33].

The use of integrated digital platforms aligns with global health system priorities, such as the Quintuple Aim, by simultaneously addressing patient and provider experience, enhancing workflow efficiency, and supporting continuity of care [14]. Evidence has shown the fragmented nature of cancer care and the high burden placed on clinicians by noninteroperable health information systems [34-37]. MyJourney’s design addresses these documented challenges by consolidating provider-facing functionality and facilitating the timely exchange of information. Platforms that coordinate tasks across teams represent a promising strategy for enhancing multidisciplinary collaboration and reducing provider workload, findings consistent with recent studies on digital navigation tools and care pathway supports [34-37]. The iterative, user-centered development process undertaken in this study further highlights the importance of interest-holder engagement in implementation research to ensure the practical relevance, adaptation, and uptake of new health technologies.

Provider-facing digital tools can reduce administrative burden when configured to local workflows and introduced through a preimplementation assessment [38-40]. Health systems should invest in interoperability and lightweight notification pathways, allowing navigators and nurses to act on results without repeatedly checking the system. Workforce implications are also notable: features such as task reminders, appointment views, and shared dashboards directly address the cognitive and administrative load of nurses and pharmacists, suggesting that digital platforms can be part of a broader strategy to sustain oncology workforce capacity [37-43]. The adaptability of MyJourney across both mature (BDC) and new (CC) implementations suggests that it has scalability potential across cancer programs and possibly to other diseases requiring similar care-delivery support. While this study focused on the provider-facing tool, the linked patient app may also reduce call volumes and enhance self-management, creating system-wide benefits.

### Strengths and Limitations

Our study had several strengths. First, our evaluation used a multimethod, iterative design that incorporated qualitative interviews and quantitative surveys at multiple stages, including the usability evaluation. Second, we engaged diverse knowledge user groups within MyJourney, including patients, nurses, pharmacists, and administrative staff, to gain a comprehensive understanding of the platform’s potential impact and areas for improvement. Third, the use of the interpretive description methodology provided detailed insights, closely grounded in participants’ experiences and clinical contexts. The integration of patient insights and provider workflow mapping into the design and rollout of the MyJourney platform at the CC helped us refine it to better align with real-world needs. Finally, interpreting our findings through RE-AIM (phase 3) offers preliminary evidence of promising implementation and potential maintenance of MyJourney, with participants able to complete core tasks and describing the app as helpful for organizing information and coordinating aspects of their care. Patients and clinicians reported perceived reductions in documentation burden and smoother workflow coordination when using MyJourney.

Limitations include a small sample confined to 2 clinics within 1 hospital, so we acknowledge that the generalizability of our findings to smaller hospitals, rural settings, or low-resource environments may be limited. However, this approach allowed us to observe both a mature implementation (ie, the BDC clinic) and an early-stage adoption (CC) of MyJourney within the same institution. Nevertheless, we acknowledge that the implementation of innovations such as MyJourney is dependent on institutional readiness, leadership buy-in, and interoperability capacity. There is also a possibility that our purposive sampling strategy to identify eligible patients, providers, and staff introduced selection bias, as our recruitment methods incorporated convenience and self-selection (eg, posters, prior consent lists, and voluntary participation by staff). This may limit the transferability of our findings. However, we included nearly all MyJourney users in our sample. Phase 2 interviews did not include oncologists or surgeons, reflecting their limited routine use of the CC Clinical Navigation Tool at this stage; future work should more directly incorporate physician perspectives, as the platform scales and physician-facing components are further integrated. Another related limitation was that the patient participants in phase 1 were predominantly English-speaking, highly educated, and reported higher income, which does not reflect the North York community. In future evaluations, we will ensure the recruitment of patients with diverse intersecting identities, including gender identity, race, language, and accessibility needs. Outcomes are primarily perceptual and descriptive at this stage of technology evaluation (particularly for describing workflow efficiency), and integration with external systems has not been tested. We did not collect objective use or long-term sustainability metrics (eg, log-in frequency and time-on-task) for the BDC implementation; therefore, our assessment of implementation “success” is based on user-reported usability, perceived utility, and integration into workflows. As such, our results should be interpreted as evidence of early implementation success rather than definitive proof of sustained adoption (ie, they should be understood as descriptive and perceptual indicators of implementation success and anticipated sustainability). Future work should incorporate objective workflow and clinical metrics, as well as measures (eg, time-motion or productivity metrics), and longer follow-up to more rigorously assess efficiency and maintenance over time.

### Recommendations for Future Research

Future work should evaluate operational outcomes such as time to key treatment steps, missed appointment rates, task completion latency, and call volumes. Studies should assess workload and burden using validated measures, paired with time-and-motion studies or log data to capture changes in work patterns. Longitudinal research is needed to track sustained use and maintenance and to understand the sustainability of MyJourney and what is required to ensure it becomes part of routine care across different clinical settings. Equity impacts warrant close attention, including experiences of patients with lower health or digital literacy and the broader accessibility of the patient app. Resource limitations prevented us from evaluating the patient app, so assessing this feature of MyJourney will be an essential step. Comparative research across different clinical settings, including rural or resource-limited sites, could also be undertaken to test MyJourney’s scalability potential. Finally, integrating use analytics from the clinical tool and the patient app with qualitative accounts will clarify mechanisms of effect and guide ongoing optimization.

### Conclusions

Results from our research demonstrated that MyJourney’s Clinical Navigation Tool may contribute to workflow efficiency, care coordination, and information management for breast cancer teams at the NYGH’s BDC and CC. The high usability scores and positive user experiences at these clinics support the MyJourney platform’s potential for scaling and integration into other settings to optimize cancer care pathways. Continued refinement of digital platforms based on user feedback and expanding integration with electronic medical records will be essential to maximize their benefit and advance high-quality, multidisciplinary oncology care.

## Supplementary material

10.2196/87973 Multimedia Appendix 1 Phase 1 interview guide.

10.2196/87973 Multimedia Appendix 2 Phase 2 interview guides.

10.2196/87973 Multimedia Appendix 3 Phase 3: evaluation surveys for the Breast Diagnostic and Chemotherapy Clinics.

10.2196/87973 Multimedia Appendix 4 Phase 1: themes and relevant quotes describing participants’ experience throughout their breast cancer journey stages.

10.2196/87973 Multimedia Appendix 5 Phase 1: features suggested by participants to include in a future patient tool.

10.2196/87973 Multimedia Appendix 6 Minimum viable feature set that was used to build the patient-facing app.

10.2196/87973 Multimedia Appendix 7 Phase 2: challenges and recommendations as reported by participants—before starting chemotherapy.

10.2196/87973 Multimedia Appendix 8 Phase 2: challenges and recommendations as reported by participants—during chemotherapy.

10.2196/87973 Multimedia Appendix 9 Phase 2: challenges and recommendations as reported by participants—end of chemotherapy and care transition.

10.2196/87973 Multimedia Appendix 10 Phase 3: usability evaluation results with representative quotes.

## Abbreviations

BDC: Breast Diagnostic Centre
CC: Chemotherapy Clinic
NYGH: North York General Hospital
RE-AIM: Reach, Effectiveness, Adoption, Implementation, and Maintenance
SRQR: Standards for Reporting Qualitative Research
SUS: System Usability Scale
