# Supplementary material for: Supporting Patients With Breast Cancer and Providers Through Treatment and Survivorship: Multimethod Implementation Study of the MyJourney Platform
Source: JMIR Cancer. 2026 Jun 10;12:e87973. doi: 10.2196/87973 (PMC13254169; doi:10.2196/87973)
Supplement: Multimedia Appendix 1 [file cancer-v12-e87973-s001.docx]

Semi-Structured Interview questions

1. Can you tell me about how you became a patient at the Breast Diagnostic Centre?
2. What was the process of you getting a diagnosis of breast cancer? Probe: discovery of lump or tumor, biopsy process.
3. Can you tell me what kind of breast cancer you were diagnosed with? Probe: understandings of the different types and stages of breast cancer?
4. Do you remember your reaction to your diagnosis?
5. What information did you receive after your diagnosis?
6. Do you remember going to a class about breast cancer at the hospital? If not – was it offered? If so – what was your experience of the class?
7. Who have you mostly been in contact with about your condition? What has that communication been like?
8. What happened after you received your diagnosis? Probe: tests, treatments, referrals, and health care professionals seen.
9. Where are you at now in your treatment plan?
10. What are the next steps for you?
11. What has the Breast Diagnostic Centre done to make your journey easier?
12. What could they do to make it better?
13. Is there anything else that you would like to mention about your experience as a patient at the Breast Diagnostic Centre?
