# Supplementary figures and images for: Supporting Patients With Breast Cancer and Providers Through Treatment and Survivorship: Multimethod Implementation Study of the MyJourney Platform
Source: JMIR Cancer. 2026 Jun 10;12:e87973. doi: 10.2196/87973 (PMC13254169; doi:10.2196/87973)

**Interview guide for Pharmacists**


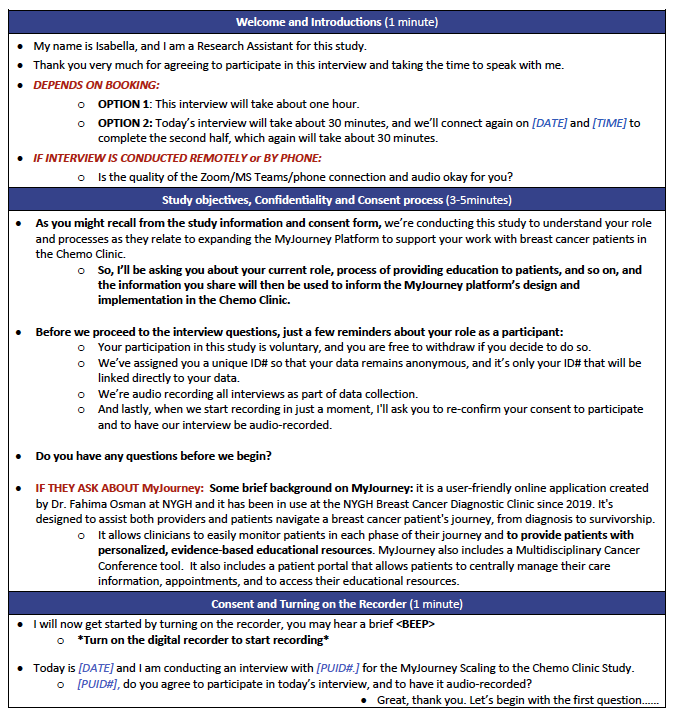


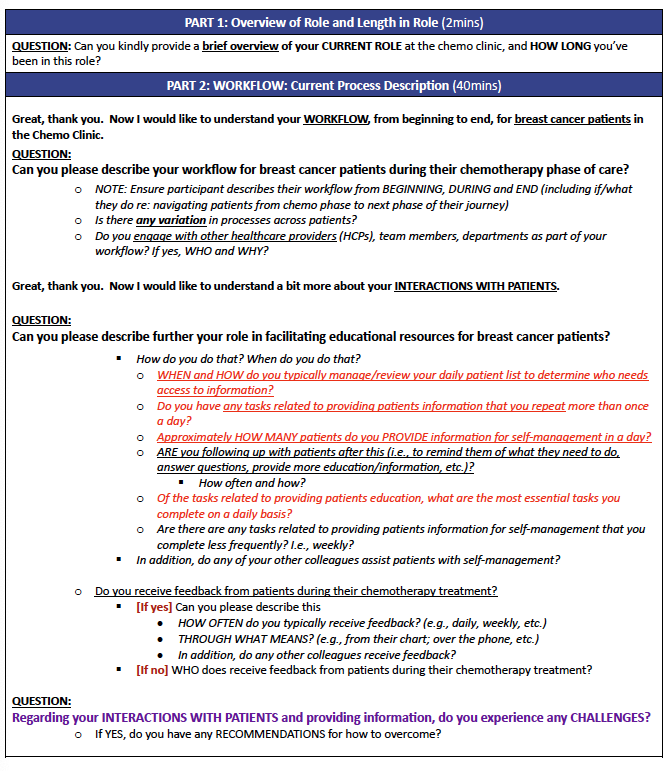


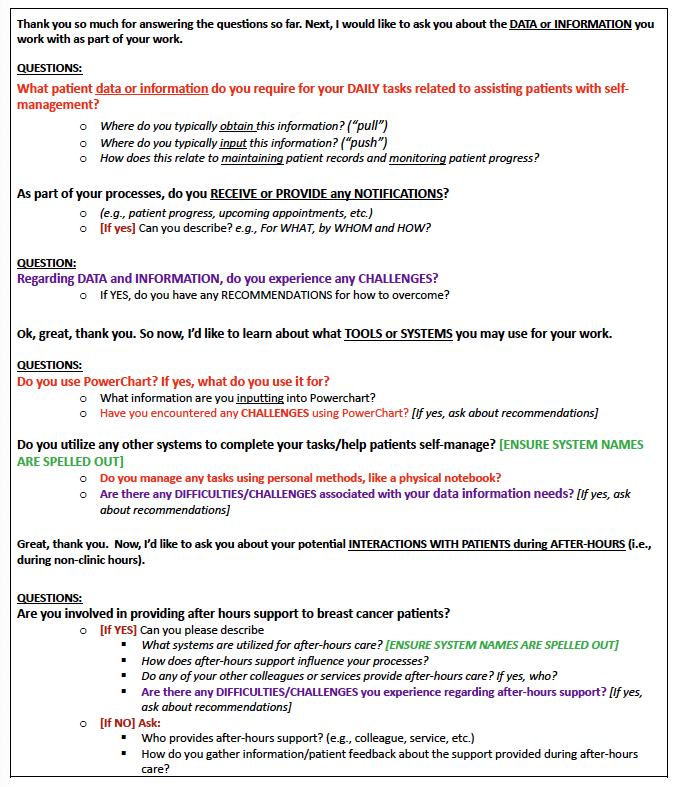


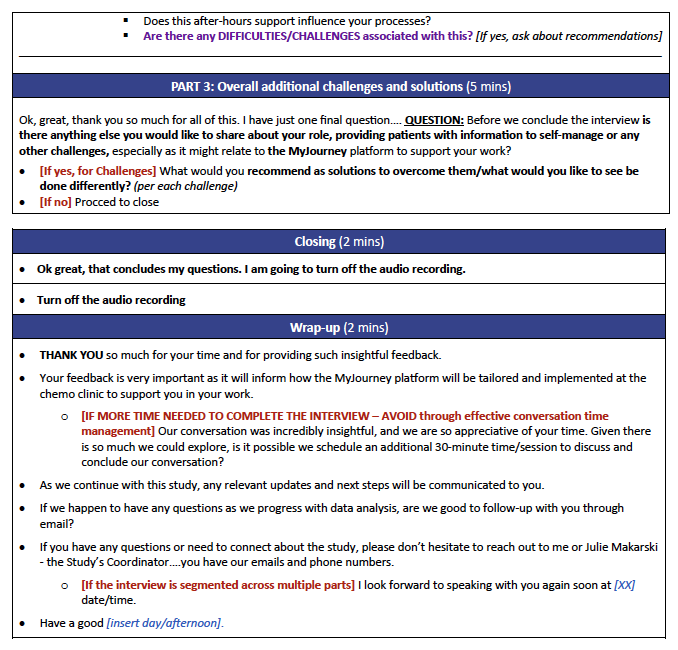


**Interview guide for nurses**

**
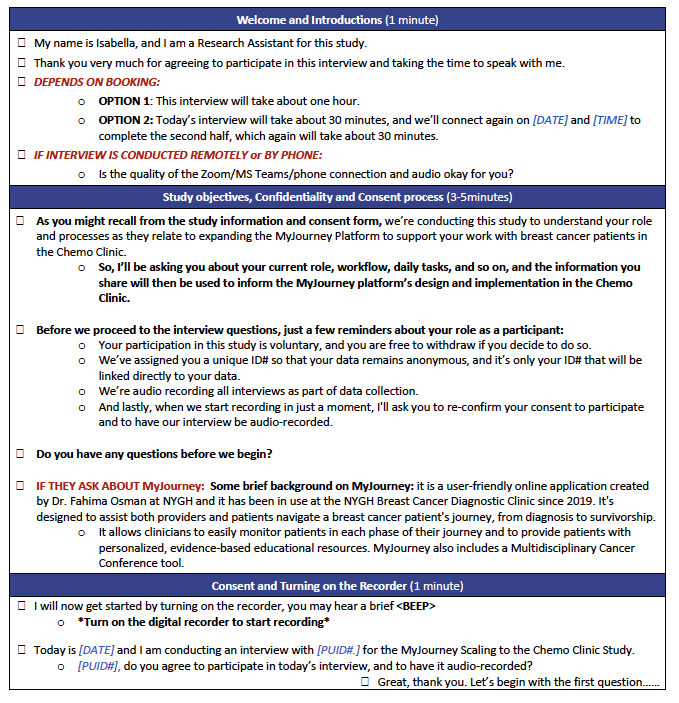
**

**
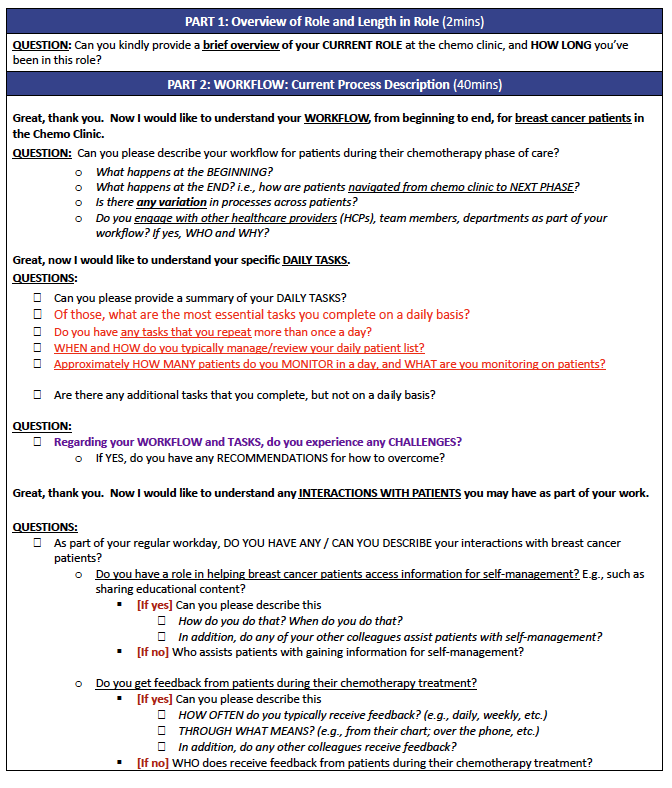
**

**
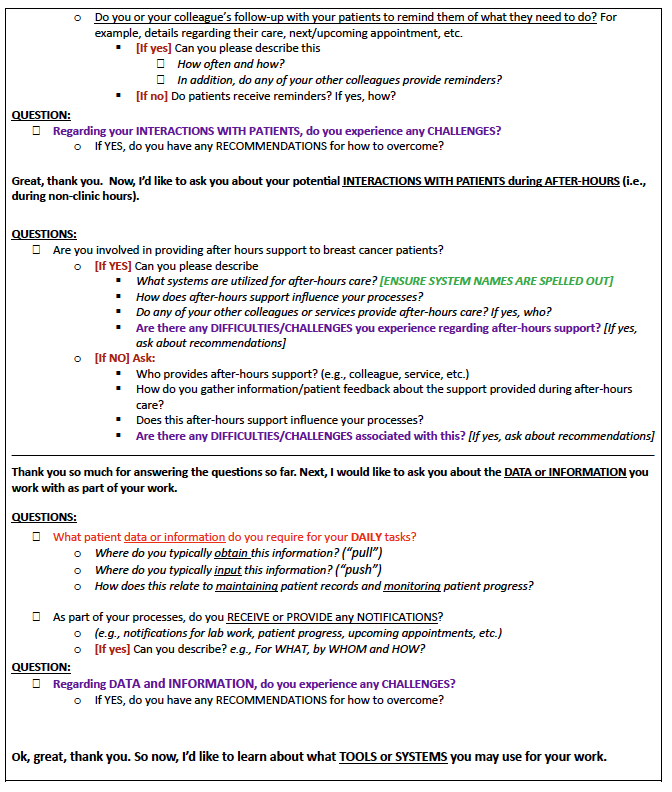
**

**
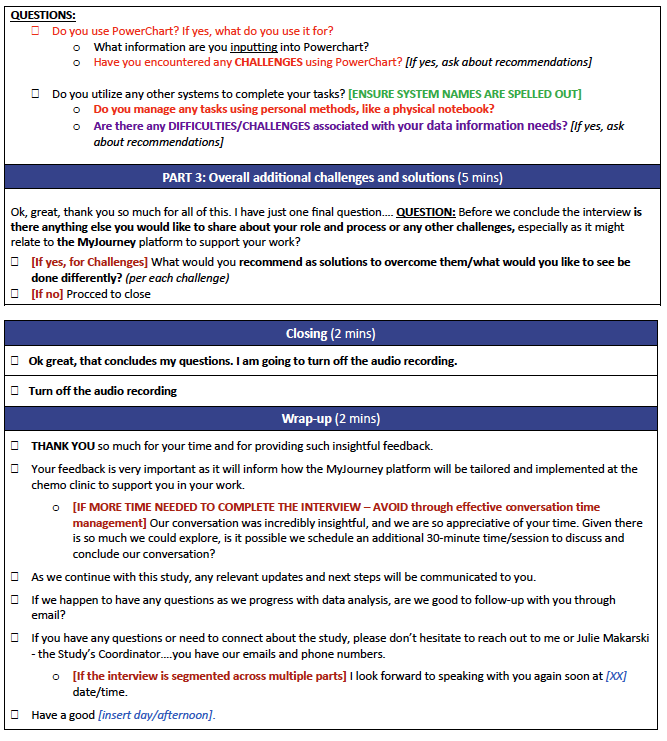
**

Supplement: Multimedia Appendix 2 [file cancer-v12-e87973-s002.docx]
