# Supplementary material for: Supporting Patients With Breast Cancer and Providers Through Treatment and Survivorship: Multimethod Implementation Study of the MyJourney Platform
Source: JMIR Cancer. 2026 Jun 10;12:e87973. doi: 10.2196/87973 (PMC13254169; doi:10.2196/87973)
Supplement: Multimedia Appendix 3 [file cancer-v12-e87973-s003.docx]

| **Survey: Breast Diagnostic Clinic** | **Survey: Chemotherapy Clinic** |
| --- | --- |
| **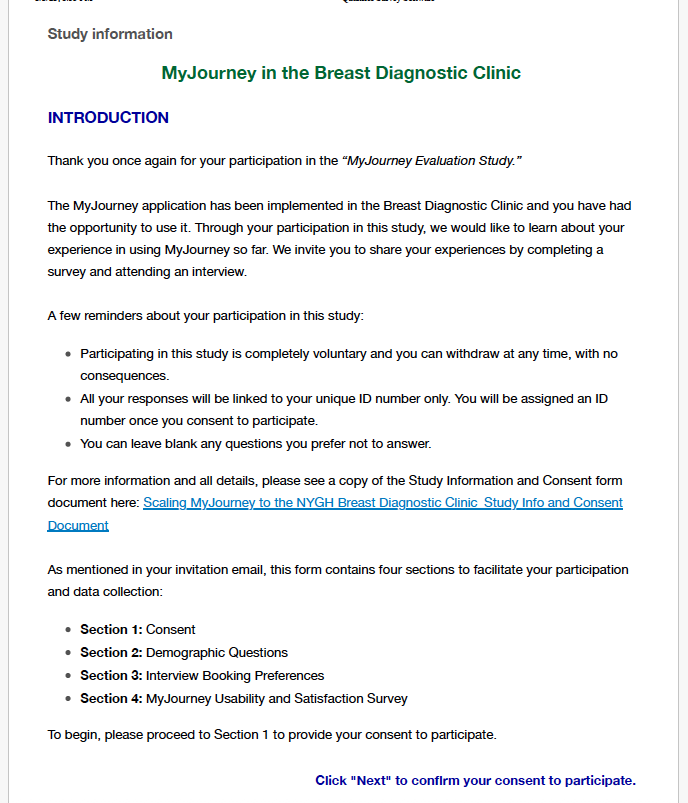** | **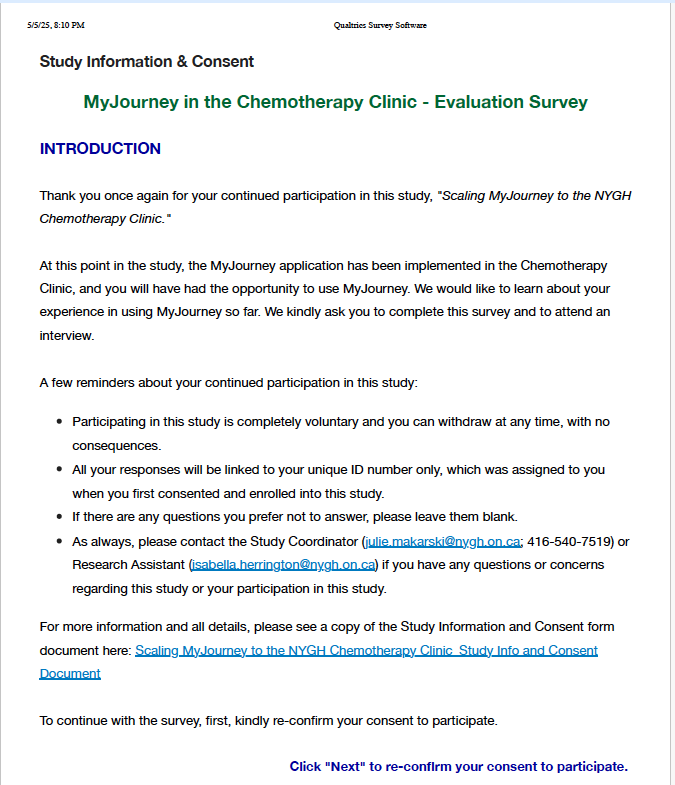** |
| **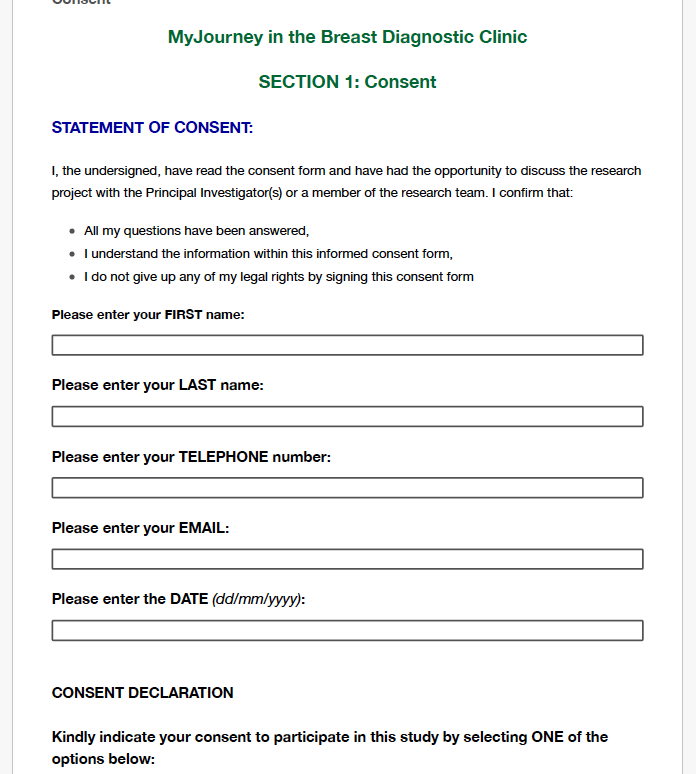** | **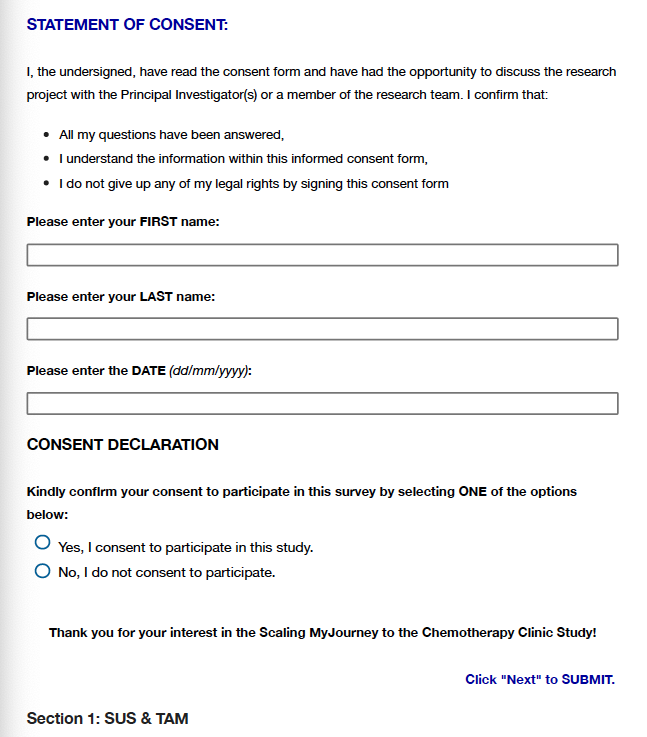** |
| **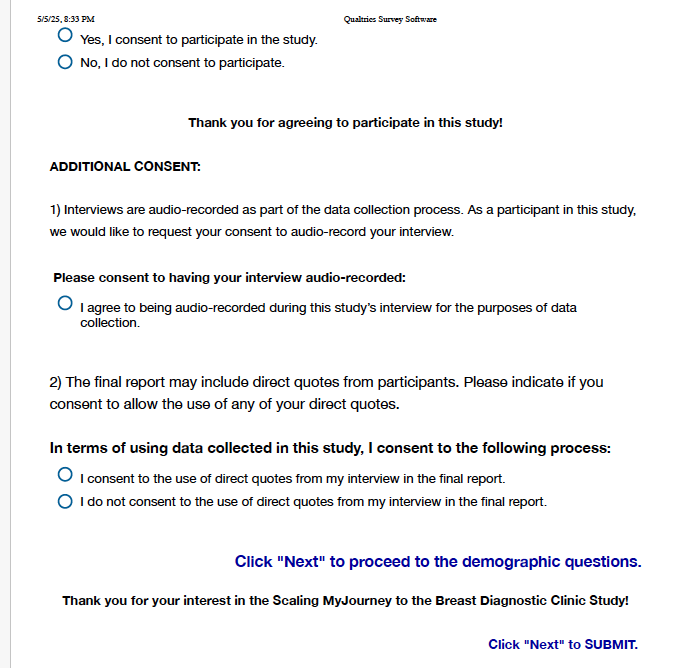** | **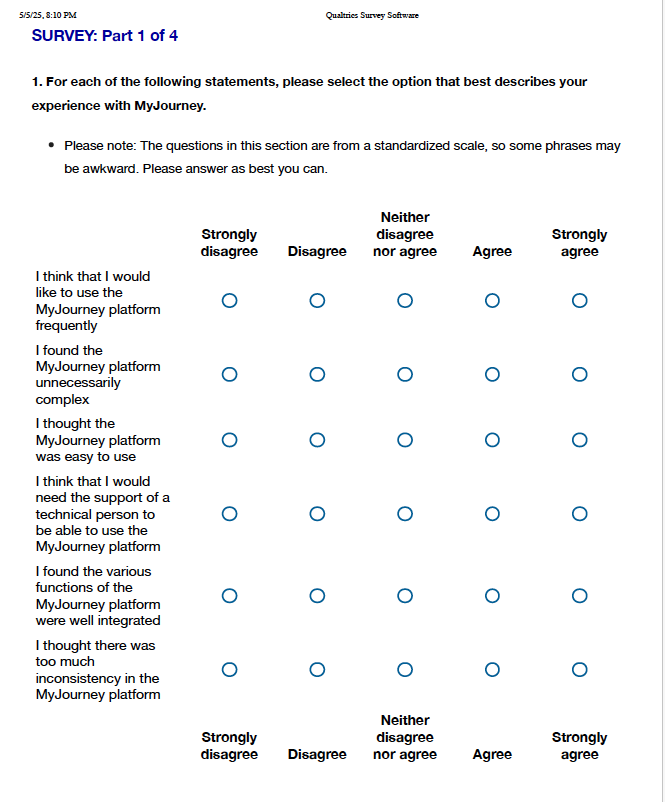** |
| **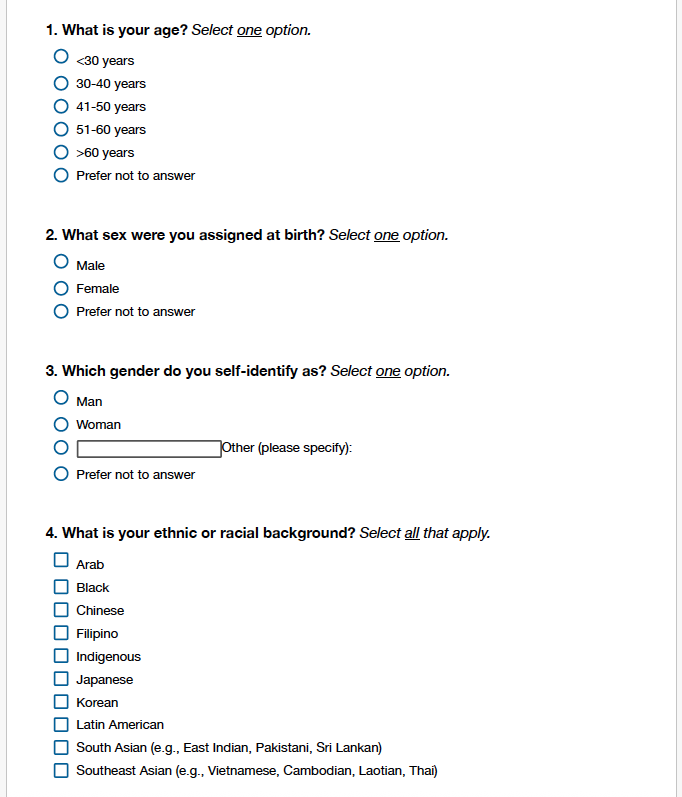** | **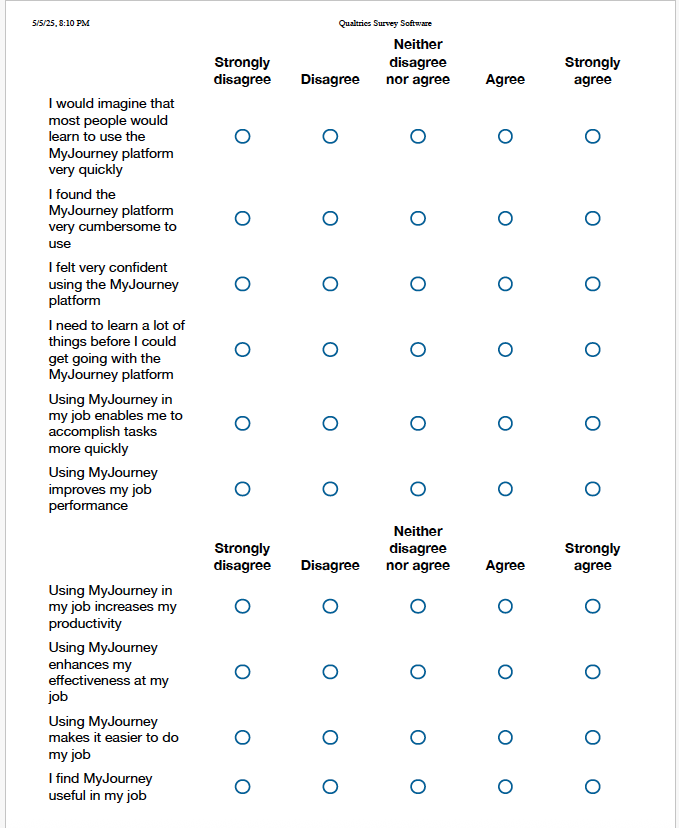** |
| **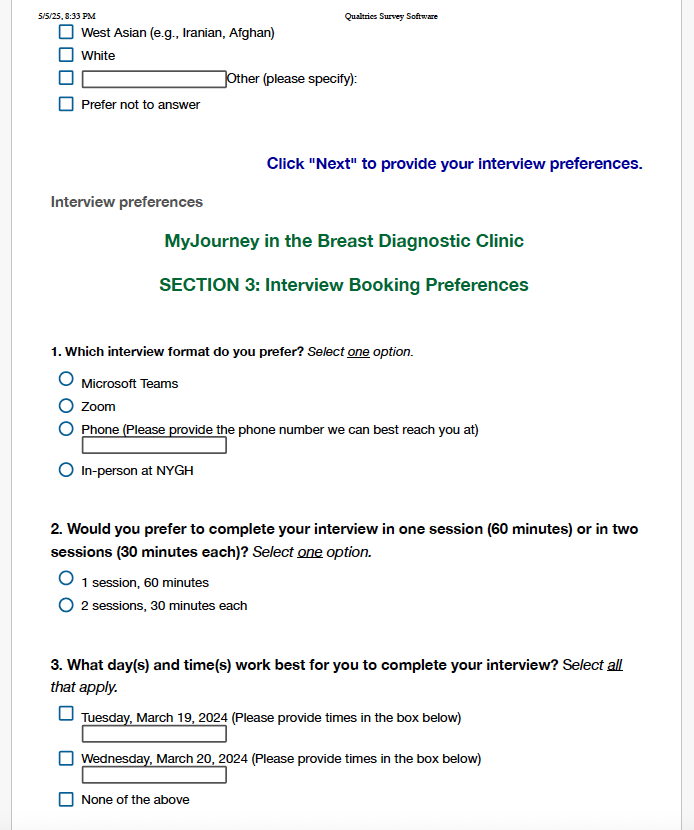** | **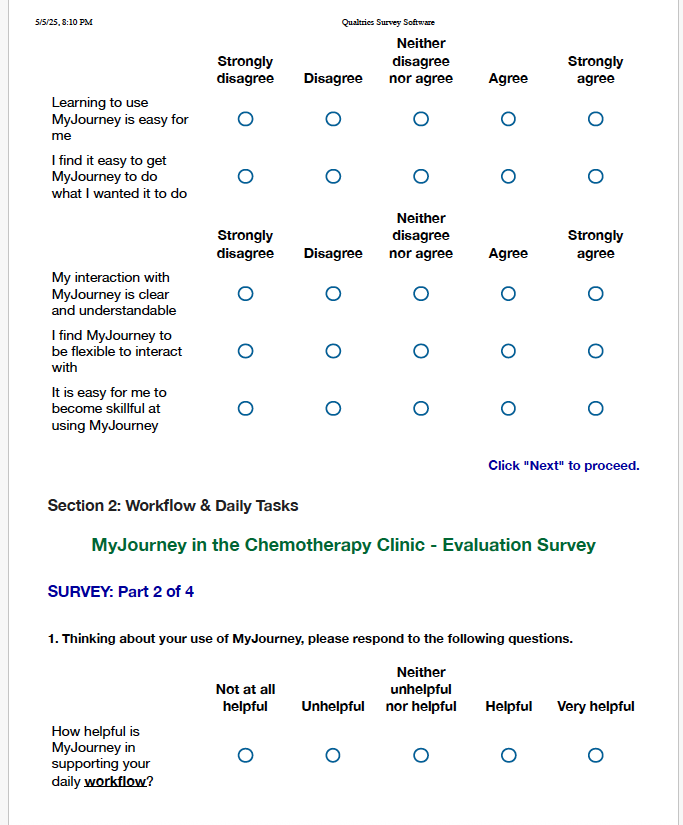** |
| **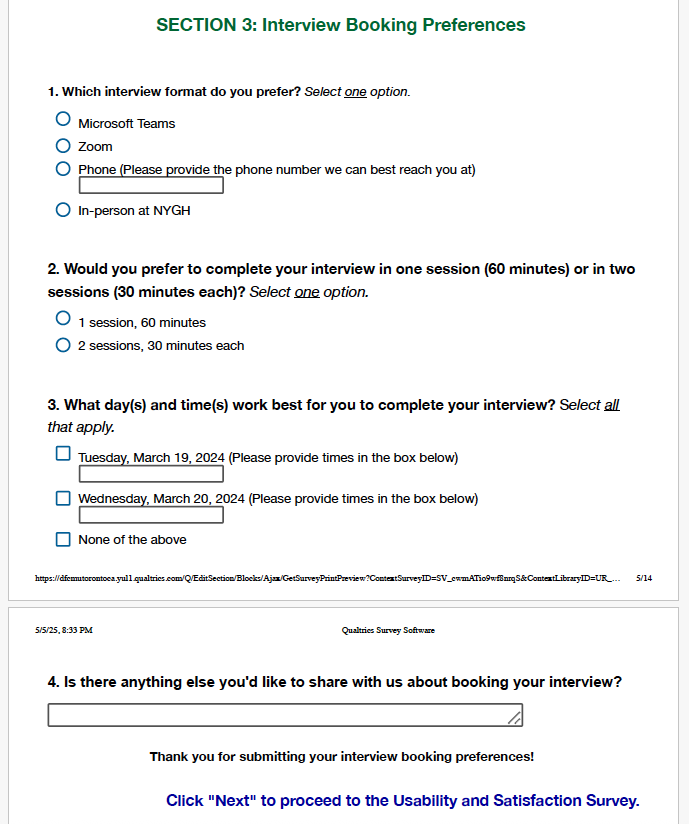** | **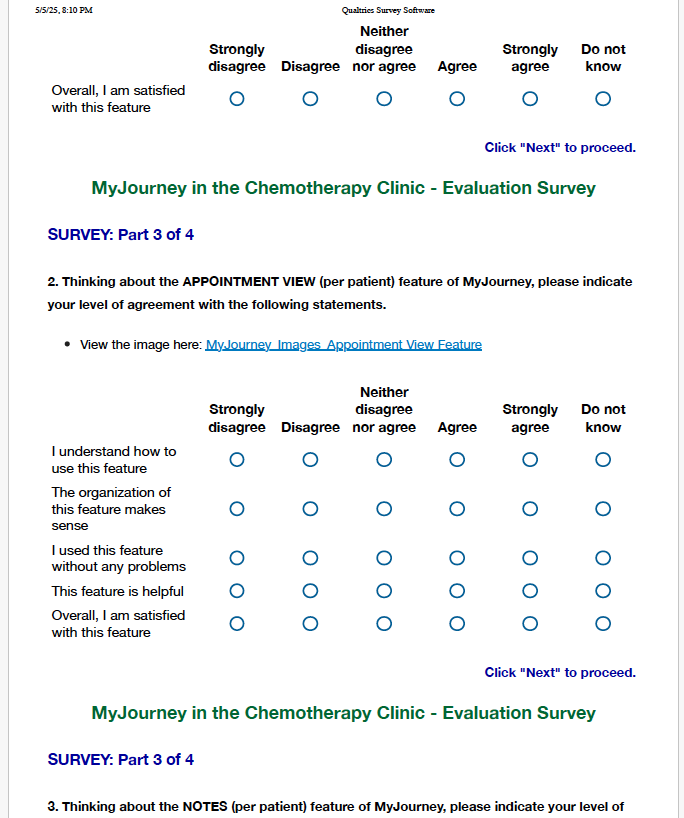** |
| **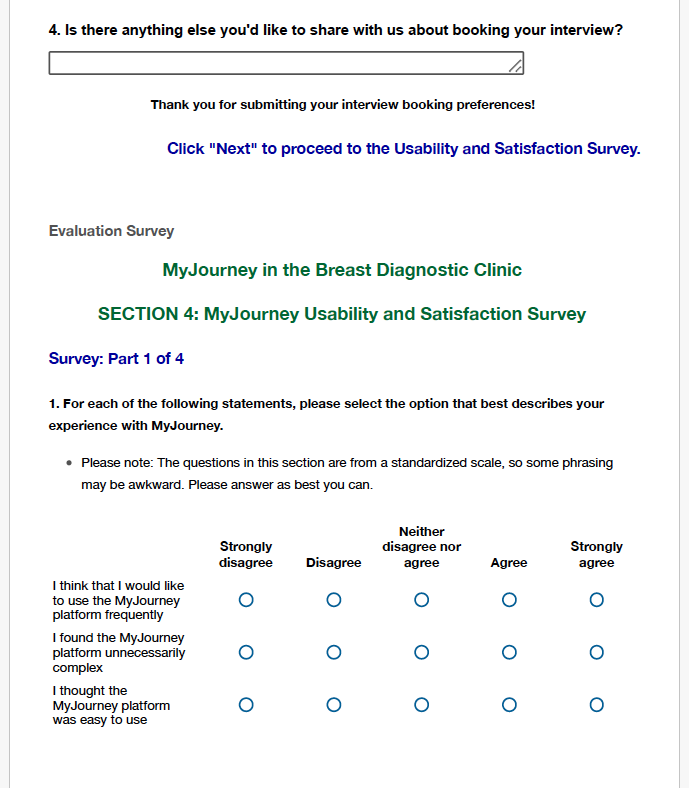** | **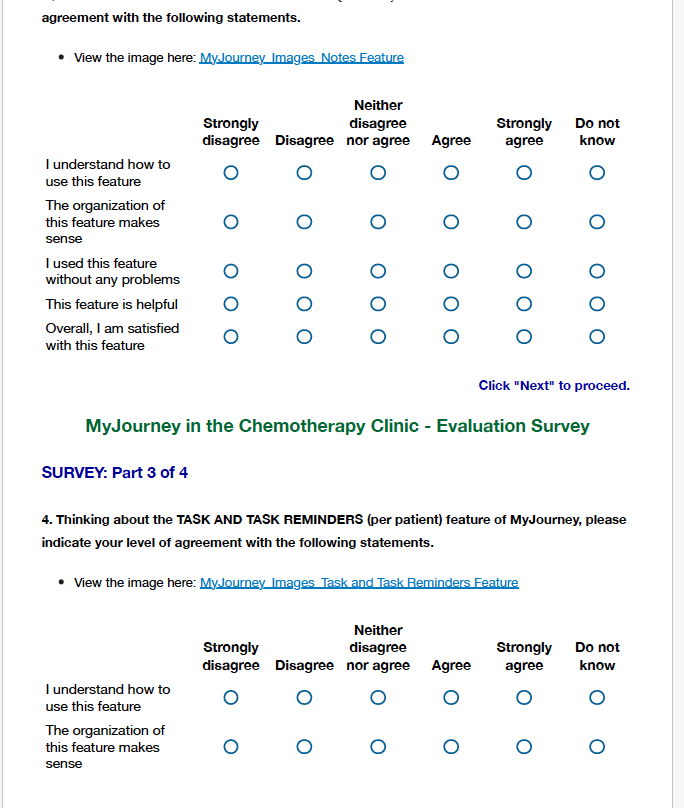** |
| **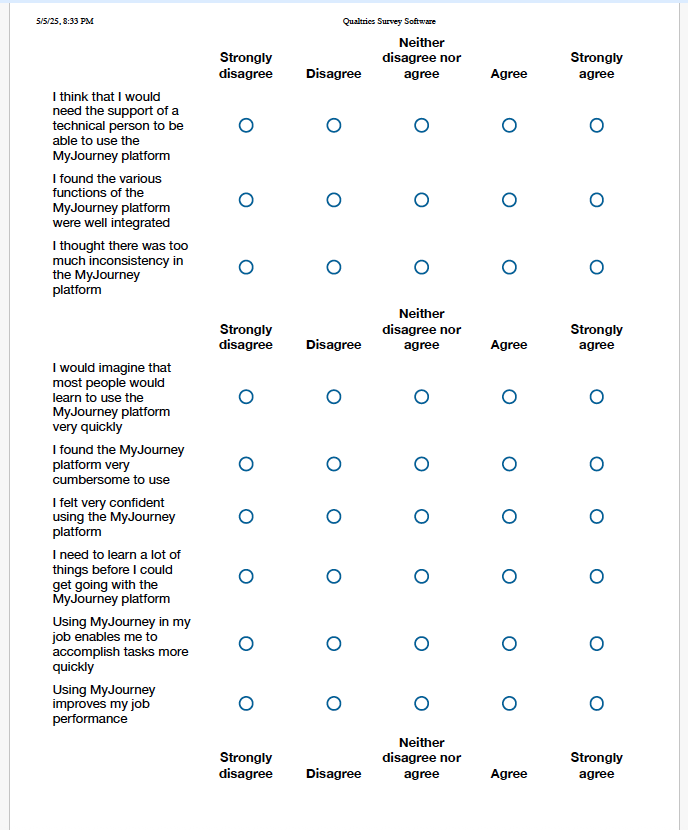** | **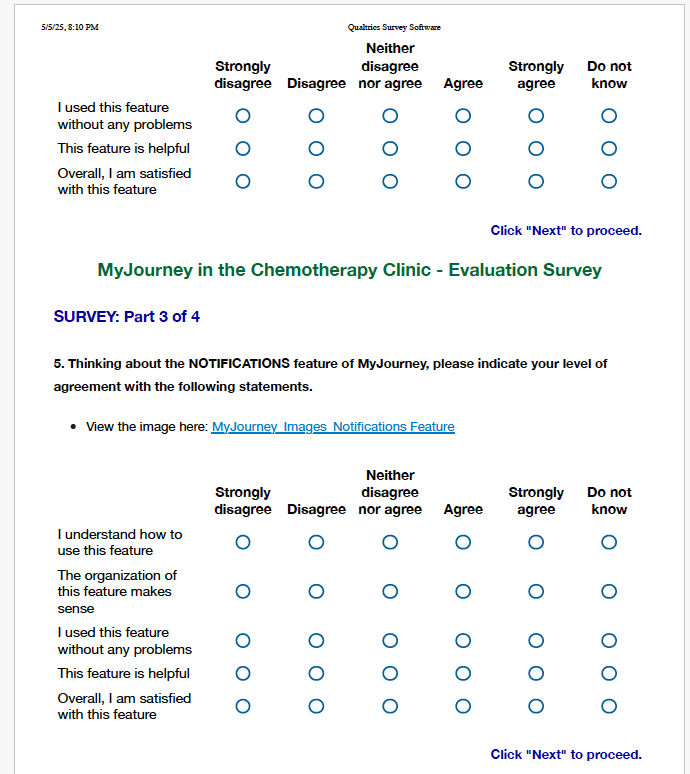** |
| **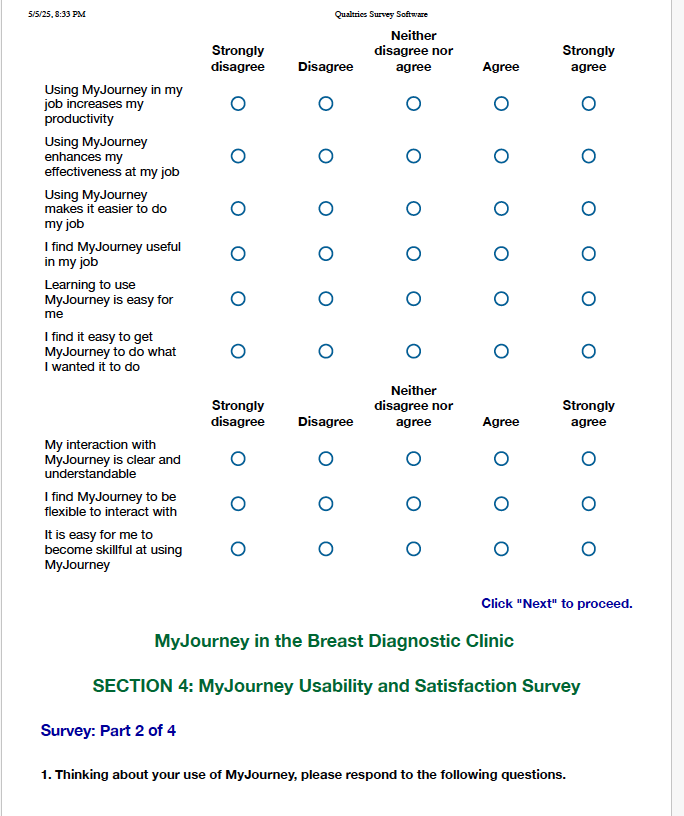** | **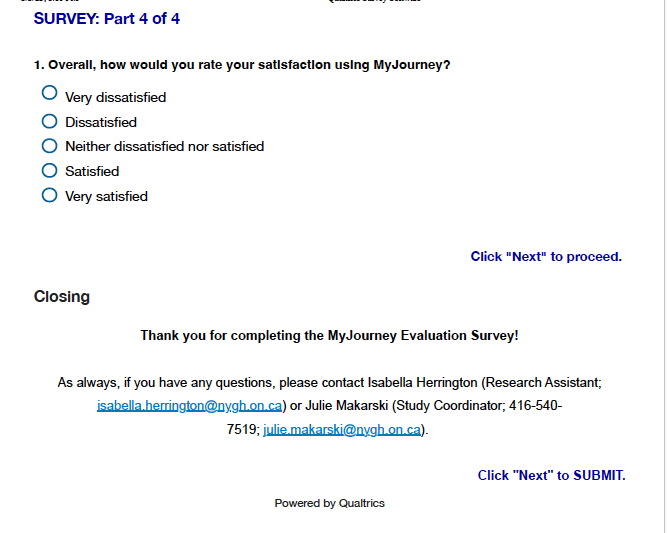** |
| **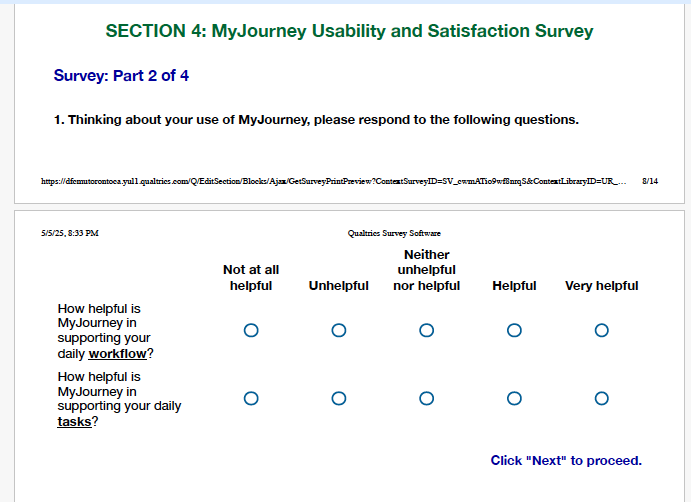** | Not applicable |
| **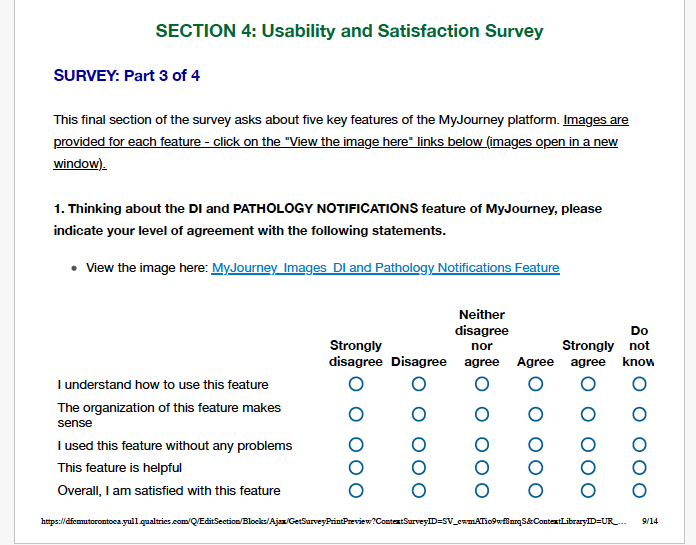** | Not applicable |
| **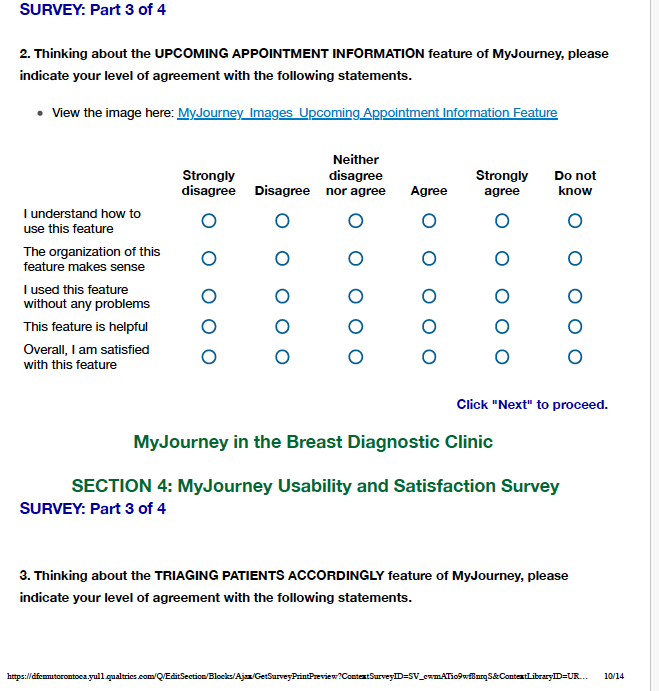** | Not applicable |
| **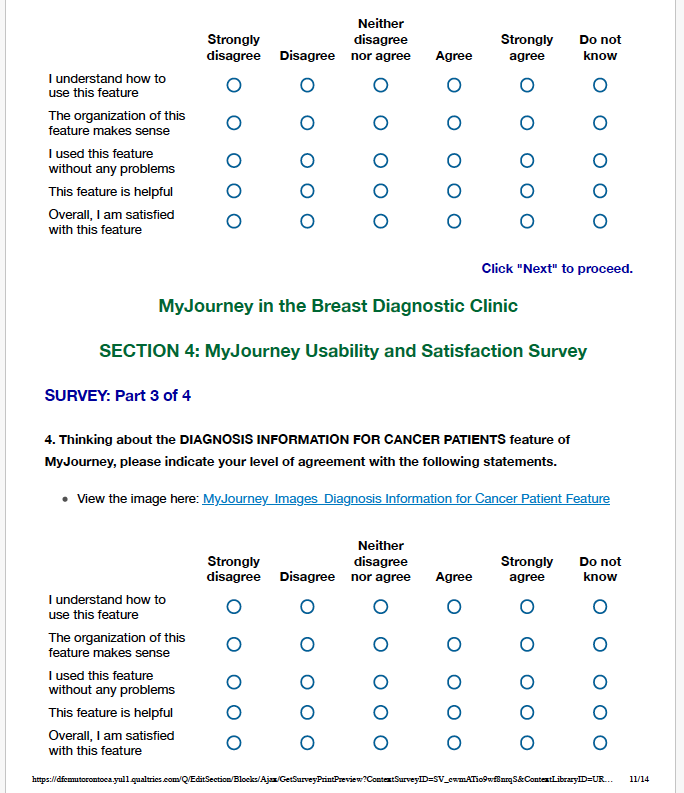** | Not applicable |
| **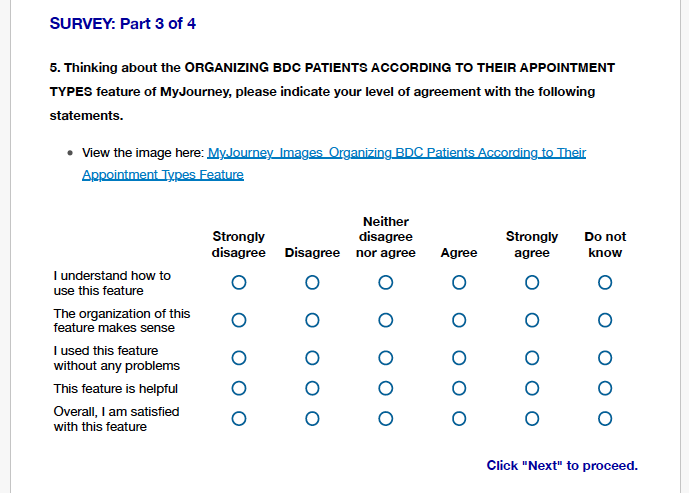** | Not applicable |
| **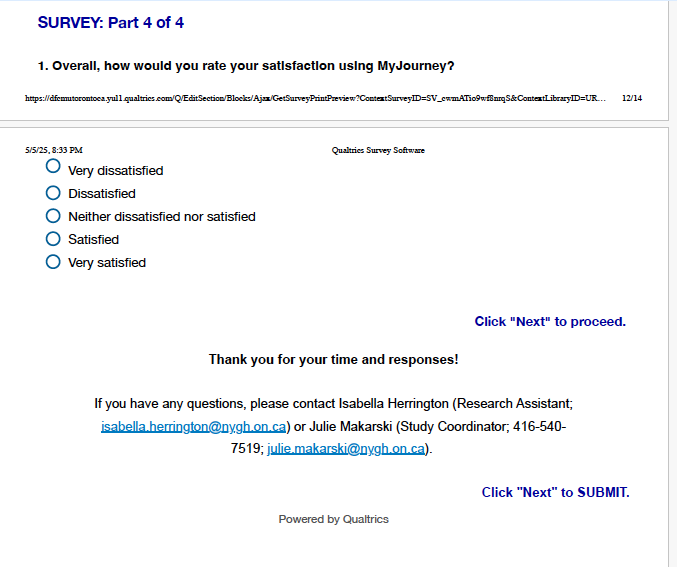** | Not applicable |
