# Supplementary material for: Supporting Patients With Breast Cancer and Providers Through Treatment and Survivorship: Multimethod Implementation Study of the MyJourney Platform
Source: JMIR Cancer. 2026 Jun 10;12:e87973. doi: 10.2196/87973 (PMC13254169; doi:10.2196/87973)
Supplement: Multimedia Appendix 4 [file cancer-v12-e87973-s004.docx]

| **Breast cancer journey stage** | **Description** | **Relevant quote(s)** |
| --- | --- | --- |
| ***Pre-diagnosis*** | Breast cancer patients typically undergo tests such as mammograms (routine screening and diagnostic), ultrasounds, Magnetic Resonance Imaging (MRIs), and breast biopsies. Participants prioritized rapid communication, clarity about next steps, and reassurance. During this phase, patients can wait one to four or more weeks to learn whether they have cancer, and if so, what type. Participants often described this phase as the most difficult of the journey. | - *I walked home (after the biopsy) and I remember it being hard to wait for the diagnosis results. This was the hardest part of the whole journey. [P7]* - *The hardest part was from when I thought I probably had breast cancer, but didn’t know for sure. I didn’t know what type, the treatment plan, what does that mean? [P16]* |
| ***During diagnosis*** | Once the type, location, and stage of the breast cancer had been determined in the pre-diagnosis phase, patients had an appointment with a breast cancer specialist – often a surgeon – to learn about their type of cancer, its unique stage (i.e., how advanced the cancer is at the time of diagnosis), and discuss treatment options. It is at this point that women begin to learn a new language and a new way of understanding their breast health. Diagnosis is situated after a period of agonizing waiting for patients, in which their futures are uncertain, and they often feel helpless. For those with a diagnosis of breast cancer, the waiting is punctuated with devastating news, described in (for most people) foreign medical terminology that exacerbates the confusion and feelings of being overwhelmed. As the treatment journey proceeds, many women begin to adopt, or at least understand, this new language. However, the diagnosis itself is a nexus of fear, confusion, and information overload.  Participants preferred a layered approach to information delivery, with reduced jargon and resources available in multiple formats. | - - *When I was diagnosed, I experienced information overload, but my husband was with me at all times to help me digest info. I took a notebook with me to help me capture info. I wish I had audio recorded these appointments. [P15]*   - *I felt overwhelmed when given information about diagnostics and treatment. It was hard to remember. I took notes in my notebook. But it was a lot to try to understand at the time. It was a lot to take in. Trying to make sense of what it meant to be positive, negative, etc. was difficult. [P9]* |
| ***During treatment*** | Diagnosis was often followed by surgery, although some women had chemotherapy before surgery to shrink the tumour size and/or control distant spread. Women had to make decisions about breast-conserving surgery or a mastectomy, and whether they wanted reconstruction surgery. On the day of the surgery, patients are asked not to drink or eat for many hours beforehand, and to be at the hospital several hours before their surgery. Many women complained about waiting and administrative confusion, which also triggered negative emotions because the waiting could be interpreted as a sign of miscommunication, indifference, error or incompetence. Participants valued consolidated care plans, timely self-care resources, and reductions in duplicative communication. | - - *The day of surgery there was massive confusion about whether I was going home or staying overnight. At the time, I got scared and thought there was a reason – there was something I wasn’t being told. Why do I have to stay? But, I didn’t have to. We waited forever, and I worried and to this day I am still worried whether that dye they put in there lasted long enough. [P4]* |
| ***In survivorship*** | - Once treatment phases are completed, most women graduate to the “survivorship” phase, where they may continue taking medications and maintain a regular schedule of imaging and follow-up appointments as part of ongoing surveillance and screening. - However, survivorship for many women means continuing to take medications with significant side effects and adapting to a new schedule of observation in which recurrence may often feel like a looming possibility. - For many women, the survivorship phase was met with confusion and a sense of abandonment. | - - *I think they need to prepare patients for after care. Your mind starts going places and you start getting paranoid and you think everything is a symptom or a sign that something went wrong, or somehow related to it.* [P9] |
|  | - Having transitioned from active treatment to more passive surveillance, some participants felt confused about “what was next” and what their role was | - *“I have no idea what is supposed to happen now. I don’t know what the plan is.”* [P4]. |
|  | - Patients were confused about what appointments and procedures were in their future, “who” their doctor was, having moved between numerous specialists during the diagnosis and active treatment phases. Part of the confusion was not having an obvious marker of being “cured” because post-treatment imaging and consultation slowly morphed into surveillance. | - There was no “graduation ceremony,” as one participant described it: *“I mean, what happens now? Who follows me? Who do I speak with?... It’s weird. There is no coordinated care. No one is in charge. It’s you. It’s very strange.”* [P18]. |
|  | - In addition, participants described wanting more resources during the survivorship phase, to provide them guidance about what they can “do” to stay healthy and prevent recurrence. - Healthcare providers perceive survivorship as a surveillance-heavy phase. - However, from a patient’s perspective, their expectations may be met more readily if it were considered a more “active” phase of the breast cancer journey. They look to their healthcare providers for guidance on how to emotionally integrate being a cancer survivor, and to provide overall health and wellness guidance to reduce their risk of recurrence and manage complications of recent active treatment. | - *“I would like recommendations about next steps as a survivor and other resources. I would like to learn how to take care of myself better post treatment.”* [P15]. |
