# Supplementary material for: Supporting Patients With Breast Cancer and Providers Through Treatment and Survivorship: Multimethod Implementation Study of the MyJourney Platform
Source: JMIR Cancer. 2026 Jun 10;12:e87973. doi: 10.2196/87973 (PMC13254169; doi:10.2196/87973)
Supplement: Multimedia Appendix 5 [file cancer-v12-e87973-s005.docx]

| **Portal Feature** | **Supporting Quote** |
| --- | --- |
| Medical records access | *"I would like a patient portal with all of my reports and bloodwork results (P6)* |
| Test results | *"Would like lab results... Takes some anxiety away when you get the results right away (P8)* |
| Appointment tracking | *"I would like my appointments in there...It’s hard to keep track of it and remember (P8)* |
| Medication information | *"Would like to see her medication lists – what drugs prescribed, when, why and the side effects” (P7)* |
| Treatment chronology | *"Would like to see the chronology of her appointments and treatment in a CareHub” (P7)* |
| Educational resources | *"I would like it to have resources, information on the type of cancer, where to get more information” (P9)* |
