# Supplementary material for: Supporting Patients With Breast Cancer and Providers Through Treatment and Survivorship: Multimethod Implementation Study of the MyJourney Platform
Source: JMIR Cancer. 2026 Jun 10;12:e87973. doi: 10.2196/87973 (PMC13254169; doi:10.2196/87973)
Supplement: Multimedia Appendix 6 [file cancer-v12-e87973-s006.docx]

| **Feature** | **Description** |
| --- | --- |
| **Integrated timeline and roadmap** | - Chronological, visual map of past and upcoming appointments, tests, and treatments (“chronology of appointments and treatments”). - A simple flow-chart style “what happens next” view, including pre-op prep and post-surgery/chemo/radiation steps, reflecting multiple comments about wanting a pathway or flow chart. - Phase-based onboarding screens at key inflection points (diagnosis, start/finish of surgery, chemo, radiation, transition to survivorship) explaining what to expect, what is normal, and who to call |
| **Results and records hub** | - Access to lab results, imaging reports, operative notes, and key letters, with clear flags for “patient-view” versions. - Plain-language explanations and “lookup” for common labs and imaging terms, responding to requests for lab result lookup and confusion about tests like oncotype and “seed lock.” - Download/print options so patients can bring records to other providers, acknowledging that patients are currently printing MyChart results for other clinicians |
| **Questions, notes and visit prep** | - Structured “visit prep” tool: lets patients note symptoms, questions, medications, and concerns before appointments, echoing people who forgot to mention medications or realized questions later. - After each visit, a quick summary (auto-generated outline plus patient-edited notes) and a space to attach photos of written diagrams, aligning with those who rely on handwritten notes and journals. - Option to upload an audio recording of the consult (if permitted) and generate key-point summaries, reflecting participants’ wish that they had recorded appointments. |
| **Secure messaging / asynchronous Q&A** | - An asynchronous “ask a question” channel (to nurse navigator/clinic) so patients who “don’t want to talk on the phone” can post questions and get replies later. - Clear routing and scope: show who receives which types of questions to reduce confusion about whether to contact surgeon vs medical oncologist vs GP. |
| **Education, resources, and “safe internet”** | - Curated, clinician-approved resource library with:   - Cancer-type specific overviews in plain language.   - Targeted content on surgery, chemo, radiation, side effects, lymphedema, fatigue, Tamoxifen, etc., addressing repeated information gaps.   - A set of “safe links” to reputable sites (e.g., BC Cancer, Canadian Cancer Society) and local supports like Wellspring, explicitly requested as a way to avoid harmful Google searching. - Phase-specific “starter packs”:   - Newly diagnosed: basics of diagnosis, staging, treatment options, how to tell family, what to bring to appointments.   - Active treatment: practical tips (e.g., black tea for hand-foot syndrome, ice for nail preservation, diet and hydration strategies), self-care suggestions (exercise, stress management).   - Survivorship: surveillance schedule, recurrence signs, lifestyle and exercise for recurrence reduction, managing fear of recurrence, navigating work/disability. |
| **Psychosocial and peer support** | - “Support finder” for psychosocial resources: local dietitians, psychologists, support groups, Wellspring classes, survivorship programs, with filters for age, parental status, and location. - Peer connection options with guardrails:   - Moderated discussion boards / Q&A threads for specific topics (e.g., “young mothers with breast cancer”) to meet the need for peers “like me” while avoiding overwhelming horror stories.   - Opt-in one-to-one peer matching, possibly mediated by a navigator, with strong content moderation guidance. - Mood tracking with gentle prompts and tips, as requested (e.g., anticipating emotional lows post-radiation or surgery), optionally shareable with clinicians or supporters. |
| **Self-management and survivorship tools** | - Symptom tracking for pain, fatigue, lymphedema, cognitive issues (“chemo brain”), and stress, with simple trend views patients can discuss with clinicians.^[[1]](#fn1)^ - Behavioural “micro-goals” (e.g., walking targets inspired by the participant who gradually increased steps), with evidence-based guidance on exercise, diet, and stress reduction. - Survivorship care plan section:   - Named lead(s) for follow-up (e.g., oncologist vs GP) to address confusion about “who is in charge now.”   - Follow-up schedule, tests and timing, and clear instructions on where to go if a new lump or symptom is found |
