# Supplementary material for: Supporting Patients With Breast Cancer and Providers Through Treatment and Survivorship: Multimethod Implementation Study of the MyJourney Platform
Source: JMIR Cancer. 2026 Jun 10;12:e87973. doi: 10.2196/87973 (PMC13254169; doi:10.2196/87973)
Supplement: Multimedia Appendix 7 [file cancer-v12-e87973-s007.docx]

| **Challenge category: sub-category** | **Challenge [Role; PUID]** | **Illustrative Quote** | **Recommendation(s); MyJourney Feature addressed? (Yes/No)** | | **Clinical context / Sub-phase** |  |
| --- | --- | --- | --- | --- | --- | --- |
| **Patient challenges** | | | | | |  |
| **Lack of access to appointment information** | Some patients miss counseling appointments due to language barriers or the severity of their illness [Pharmacist; P08] | *“It may be a language barrier for some patients, and they don't get it; for others, it may be the acuity of their illness, and they're not able to come. So, I think that there's kind of a mixed bag, and I don't know if there's any one reason why they don't show up and one bulletproof solution for it either”* | No recommendation provided; Solution 2: Upcoming/Past Appointments View | | Before counseling appointments | |
|  | On average, patients miss their scheduled counseling appointments 10-20% of the time [Pharmacist; P08] | -- | No recommendation provided; Solution 2: Upcoming/Past Appointments View | |  |  |
|  | Patients occasionally miss their appointments, necessitating virtual counseling sessions or rescheduling for the next treatment day. A virtual session is less ideal for patient understanding and support [Pharmacist; P08] | -- | No recommendation provided; Solution 2: Upcoming/Past Appointments View | |  |  |
| **Early arrival times for appointments** | Some patients arrive significantly earlier than their scheduled appointment time and want to be seen early [Pharmacist; P08] | "Another challenge is sometimes patients come a lot earlier for their appointment. They go, 'Well, I came for my blood work. I'm here now. Can you see me now?'" | No recommendation provided; Solution 2: Upcoming/Past Appointments View | |  |  |
| **Lack of access to appointment information** | Patients frequently inquire about their doctor's appointment times with pharmacists daily, who do not have access to this information. Patients then need to wait for this information or ask someone else [Pharmacist; P05] | "Let's say... I'm talking to a patient about treatment, and she asks, 'Is that when I'll see Dr. [X]?' and I say, 'I have no idea.' The oncologists' follow-up appointments are completely separate from what we can see in Cerner" | Provide Clin One pharmacist with access to patient oncologist appointments to better assist with appointment reminders and coordination; Solution 2: Upcoming/Past Appointments View | | During counselling appointments | |
| **Privacy concerns** | Some patients may refuse to receive resources due to privacy concerns with sharing their email address [Pharmacist; P08] | -- | No recommendation provided; Solution 5: Educational Content via Mobile App | |  |  |
| **Lack of technology familiarity, literacy and access** | Patients' access to educational videos may vary depending on their familiarity with technology or access to computers, particularly for older adults [Pharmacist; P02] | -- | No recommendation provided; Solution 5: Educational Content via Mobile App | |  |  |
| **Administrative and process challenges** | | | | | | |
| **Incomplete information:** Missing information | Prescriptions are sometimes missing in Kroll and lack an oncologist's signature, requiring intervention by a drug access navigator. Perception that this issue originates from the oncologist [Pharmacist; P08] | -- | No recommendation provided; No | | Before counseling appointments | |
|  | Information about the patient's regimen or prescribed treatments is sometimes missing from the patient scheduling list [Pharmacist; P02] | "Scheduling…only about half the time does it include the regimen or treatments the patient is starting" | Patient appointment information should consistently include details about the patient's treatment regimen to ensure easy lookup; Solution 1: Treatment Summary | |  |  |
| **Incomplete information:** Not updated | Patient treatment schedules may not be updated accurately. Consequently, Clin Two pharmacists may not be aware of changes until the day of the patient's treatment, making counseling preparation difficult [Pharmacist; P05] | "The biggest challenge…right now, it's a manual report with pharmacists going line by line… It's taking a lot of inferences [to determine who needs counseling] because scheduling is inconsistent" | (i) Provide real-time, accurate information when treatment plans change (e.g., switching from chemo A to B); (ii) Integrate Clin One's counselling reports in a central dashboard that clearly indicates which patients have been previously counseled and which are new; (iii) Implement a filter or automated list to quickly identify new patients needing Clin Two counselling; (iv) Schedule appointments in a consistent way, avoiding free-text entries that require pharmacists to make inferences about new patient status; Solution 2: Upcoming/Past Appointments View | |  |  |
| **Notification challenges:** Form of notification | The notification process for Clin Two pharmacists to counsel patients is less straightforward than for Clin One pharmacist [Pharmacist; P05] | *“It is quite a challenge. I must say, it's not as straightforward as the Clin One Pharmacist [for becoming aware a patient needs counseling] because…we don't know the patient needs to be counseled until the nurse sends a notification to say, ‘This patient needs to be counseled. Looks like they didn't get counseling by the Clin One’ So, it's pretty ugly.”* | Provide real-time, accurate treatment information, including updated appointment schedules from the patient's doctor. Ensure clear visibility of which patients have been counseled by Clin One and which are new. Additionally, implement a filter to quickly identify new patients from the full list; Solution 2: Upcoming/Past Appointments View | |  |  |
| **Notification challenges:** Timing of notification | Clin Two pharmacists occasionally receive patients with early morning appointments on short notice, complicating preparation for counseling [Pharmacist; P05] | "For clinical two pharmacist, we don't know the patient needs to be counseled until the nurse sends a notification the [day of " | No recommendation provided; Solution 2: Upcoming/Past Appointments View | |  |  |
|  | Perception that Clin One pharmacists review funding too late in patients’ journeys, which can lead to delays in patient treatment [Pharmacist; P07] | "We often identify funding issues too late…sometimes when the patient is already in the chair…Even if it's caught the day before, it's still too late" | Improve communication between pharmacists and physicians by notifying pharmacists earlier when the physician makes the treatment decision; Solution 2: Upcoming/Past Appointments View;  Solution 4: Tasks Reminders for Users on a Patient Level | |  |  |
| **Workload management:** Manual processes and preparation | Medication templates must be manually customized for each patient based on their treatment schedule and regimens, which can be complex [Pharmacist; P02] | *“So, we have standard supportive care medications, but sometimes the physician might make adjustments, so we have to tailor the schedule for the particular patient”* | Have medication templates available for different treatment schedules so pharmacists don't have to make one from scratch; No | |  |  |
|  | Manually updating patient medication charts with each change in chemotherapy is time-consuming for pharmacists [Pharmacist; P05] | -- | Automate the process of creating new medication charts when patients’ treatment regimens are updated; No | |  |  |
|  | Developing medication templates requires a lot of printing, which was perceived as an outdated practice [Pharmacist; P06] | *“Unfortunately, I still depend on printing quite a bit…to help with Clin One or Clin Two activities”* | Provide Clin One and Clin Two pharmacists with tablet devices to view the templates and access information during counseling appointments; Solution 5: Educational Content via Mobile App | |  |  |
| **Communication challenges:** Differing communication delivery methods | There are delays in response times when communicating with oncologists using Teams due to factors such as, workload or availability [Pharmacist; P08] | *“Sometimes we have a big gap in time between us sending the request or the question and the response from the oncologist”* | Consider adding primary care nurses to assist oncologists at the clinic with managing their workload more effectively; No | | After counselling appointments | |
| **Workload management:** Task volume and variability | The workload and task variety of Clin Two pharmacists makes answering patient questions and follow-ups difficult [Pharmacist; P07] | -- | No recommendation provided; No | |  |  |
| **Health information technology system challenges** | | | | | |  |
| **Cerner PowerChart:** Patient data inaccessible | Paper-based reports, such as the genetic testing marker DPYD, must be scanned into Cerner PowerChart, occasionally causing delays [Pharmacist, P02] | *“Right now, over 90 percent of our documentation or the reports and stuff are electronic, but then there are certain isolated reports that are still like in paper, so those will be scanned in. So, one example would be like a specific genetic testing marker called DPYD…Sometimes there can be delays with the scanning process. We don't get to view it in real time. We might have to chase down those results”* | No recommendation provided; No | Before counseling appointments | |  |
|  | Cerner PowerChart does not sync with physicians' private office systems, making their notes inaccessible to nurses [Pharmacist; P06] | "You may not be able to find the information you need in a timely fashion…notes from external offices don’t come across" | No recommendation provided; No |  |  |  |
| **Cerner PowerChart:** Patient information is not comprehensive | Pharmacists cannot access patient-related information outside of PowerChart or Connecting Ontario, such as treatments prescribed outside the hospital setting, notes from physicians' private offices, and non-government (ODP) funded medications [Pharmacist; P07] | *“The other issue that we have at our center…, but our physicians have private offices. And we don't have access to those in their private offices, so that is a huge, huge challenge for us. We cannot see dictations…We cannot see prescriptions that were written from their offices”* | No recommendation provided; No |  |  |  |
| **Cerner PowerChart:** Usability | Cerner PowerChart lacks user-friendly filtering, making it difficult for pharmacists to quickly find key information in notes [Pharmacist; P07] | "Anytime someone leaves a note, the heading doesn’t specify what’s in it…It's not user friendly" | No recommendation provided; Solution 3: Customized Notes, Tasks and Reminders for Users |  |  |  |
|  | Navigating multiple areas within Cerner PowerChart is complex, making it challenging for pharmacists to find necessary information quickly [Pharmacist; P06] | *“Powerchart or Cerner is a pretty complex system. There are many buttons, many areas…Sometimes you may not be able to find the information you need in a timely fashion”* | No recommendation provided; Solution 1: Treatment Summary |  |  |  |
|  | The administrative assistant seems to be unaware of how to access notes in Cerner about funding delays in scheduled appointments, leading to patients being rescheduled to see a pharmacist sooner than intended [Pharmacist; P07] | -- | No recommendation provided; Solution 1: Treatment Summary;  Solution 2: Upcoming/Past Appointments View;  Solution 3: Customized Notes, Tasks and Reminders for Users |  |  |  |
| **Connecting Ontario:** Patient information is not comprehensive | The availability of patient medication data in Connecting Ontario varies by location and does not include medications not funded by the Ontario Ministry of Health, such as over-the-counter drugs, supplements, patient-paid medications, and those covered by private insurance [Pharmacist; P05] | -- | No recommendation provided; No |  |  |  |
|  | Family doctors’ dictations do not feed into Connecting Ontario [Pharmacist; P05] | -- | No recommendation provided; No |  |  |  |
| **Limited system interoperability** | OPUS is not interfaced with Cerner, requiring manual transcription of prescription orders, which is time-consuming and prone to errors [Pharmacist; P02] | *“Because OPUS is not interfaced with Cerner, so it's a standalone system…The electronic order has during order entry we have to actually transcribe that. There is a risk for errors when we are transcribing or re-entering stuff”* | No recommendation provided; Solution 1: Treatment Summary |  |  |  |
|  | Clin One pharmacist must access PowerChart and the OPUS order entry system to review details of the patient's regimens [Pharmacist; P05] | -- | Have a centralized dashboard that includes all relevant information (i.e., the doctor's name and the patient's treatment regimen) and communication in one place for Clin One pharmacist; Solution 1: Treatment Summary |  |  |  |
| **Cerner PowerChart:** Restricted access and permissions | Pharmacists are unable to view oncologists' appointments in Cerner PowerChart, limiting their ability to remind patients of upcoming appointments [Pharmacist; P05] | "Let's say... I'm talking to a patient about treatment, and she asks, 'Is that when I'll see Dr. [X]?' and I say, 'I have no idea.' The oncologists' follow-up appointments are completely separate from what we can see in Cerner" | Provide Clin One pharmacist with access to patient oncologist appointments to better assist with appointment reminders and coordination; Solution 2: Upcoming/Past Appointments View | During counselling appointments | |  |
| **Cerner PowerChart:** Difficult and time intensive data entry | Cerner PowerChart requires Clin One and Clin Two pharmacists to manually input all documentation, which is time-consuming and inefficient [Pharmacist; P05] | "I think the greatest challenge is that it is not the most efficient way for reporting…There's a lot of point and clicks. It's a very cumbersome system. Not as easy as I'd like to actually document on" | (i)Pre-populate medications in Cerner PowerChart from an existing database to avoid manual entry; (ii) Implement voice-to-text for documentation interactions with patients; (iii) Reduce excessive pointing and clicking in Cerner to streamline navigation; No | After counselling appointments | |  |
|  | Accessing and inputting information in PowerChart is time-intensive [Pharmacist; P08] | -- | No recommendation provided; Solution 1: Treatment Summary |  |  |  |
| **Limited system interoperability** | The Clin One pharmacist must navigate between several applications, simultaneously to prepare for patient counseling sessions [Pharmacist; P05] | *“Yeah, there's no doubt that if everything was pulled together in one spot with a nice quick screen to show everything, that would be really ideal, because right now, we're just having to juggle so many different spots at one time”* | Have a centralized dashboard that includes all relevant information (i.e., the doctor's name and the patient's treatment regimen) and communication in one place for Clin One pharmacist; Solution 1: Treatment Summary | Before and after counselling appointments | |  |
| **Health resource challenges** | | | | | |  |
| **Complexity of**  Funding | Pharmacists face pressure to accurately verify funding for patient treatment, as the hospital must cover costs not funded by the Ministry of Health [Pharmacist; P07] | -- | No recommendation provided; No | Before counseling appointments | |  |
| **Human resources:** Patient-to-nurse ratios | The complexity of patient treatments, increasing patient volume, and requests for expedited care pressure nurses to complete tasks quickly despite limited resources and staff [Pharmacist; P06] | *“Treatments are getting more complex…this impacts things like staff resources, chair time, and the management, and the funding piece as well…So there's always that kind of pressure…And I think by and large, people are feeling the stress of that”* | No recommendation provided; No |  |  |  |
| **Human resources:** Limited staff availability | Contacting oncologists when they are not physically present at the clinic or working on the day of the patient's treatment is challenging due to differing schedules [Pharmacist; P02] | *“The treating oncologist is not always physically in the building…but patients can be scheduled on their non-clinic days…making it more challenging to reach them”* | No recommendation provided; No |  |  |  |
| **Limited physical space** | The Clin One counseling space, shared with the vaccine clinic, can be distracting for patients, especially during sensitive counseling sessions [Pharmacist; P06] | "The physical location of the counseling area…is still a shared space…a pretty significant distractor, especially when discussing sensitive topics" | No recommendation provided; No | During counselling appointments | |  |
| **Human resources:** Division of responsibilities | The rotation of Clin One and Clin Two pharmacists complicates communication and tracking of patient counseling, risking the loss of critical information during patient handovers among healthcare providers [Pharmacist; P07] | *“Clinical Two, it is a rotating position…hard to know what your colleague did the day before…challenging to pick up where they left off"”* | No recommendation provided; Solution 1: Treatment Summary;  Solution 3: Customized Notes, Tasks and Reminders for Users; Solution 4: Tasks Reminders for Users on a Patient Level | After counselling appointments | |  |
|  | Clin Two role, compared to Clin One has to "clean sweep" or address issues that were missed, meaning they are handling large number of new patients and many questions which can be overwhelming [Pharmacist; P08] | *“See, because it's [Clin Two’s role] is a catch-all, like you're catching—you're like the sweep, right? The clean sweep. So whatever was missed before, you're picking up the pieces. It's such a hard job because one day you may be, like, really like the other day. You're running because not only do you have a lot of new patients, but you've got a lot of questions, whatever. So it's a tough one”* | No recommendation provided; Solution 1: Treatment Summary;  Solution 3: Customized Notes, Tasks and Reminders for Users; Solution 4: Tasks Reminders for Users on a Patient Level |  |  |  |
| **Quality of care challenges** | | | | | |  |
| **Patient education and counselling:** Non-tailored resources | Medication charts lack pictures, can only be printed in black and white and only available in paper format, which limits patient understanding, especially for patients whose English is not the first language [Pharmacist; P05] | "Medication charts…could be more visual…color would be great…regardless of format, they’re not available electronically" | (i)Make the charts more visual by adding pictures of medications so patients can identify their medications; (ii) Provide colour printing options so it's readable; (iii) Make charts available electronically so patients can access on their phones at any time; (iv) Standardize the charts through a forms process, so they're the same document being given to patients; Solution 5: Educational Content via Mobile App | Before counseling appointments | |  |
| **Appointments:** Inconsiderate wait times for patients | Patients sometimes experience long waits for their Clin One counseling appointments if they arrive early for other appointments, such as bloodwork, indicating a scheduling gap and causing them to be in the hospital longer than needed [Pharmacist; P06] | *“Patients might come in for blood work and then for counseling later… it doesn't make sense that they have to hang around for a few hours…So, there may be opportunities to improve on the scheduling”* | No recommendation provided; No |  |  |  |
| **Patient education and counselling:** Inconsistencies | Pharmacists may not consistently offer IMD Health education videos to patients as they anticipate some patients saying no due to privacy concerns [Pharmacist; P08] | "The problem with the IMD Health videos is…even when we do offer it, some patients say, ‘No…because you'd have to use their email address’" | No recommendation provided; Solution 5: Educational Content via Mobile App | During counselling appointments | |  |
| **Patient education and counselling:** Poor timing of patient education | Clin Two pharmacists face challenges with the timing of patient education, as clinical issues or knowledge gaps may not be identified until the patient begins chemotherapy, potentially leading to treatment discontinuation [Pharmacist; P05] | -- | No recommendation provided; Solution 5: Educational Content via Mobile App |  |  |  |
| **Patient education and counselling:** Time constraints | Clin One pharmacist’s face challenge due to the one-hour time limit for scheduled counseling sessions, often resulting in incomplete sessions that require follow-up by Clin Two pharmacists [Pharmacist; P07] | "At least 50% of the time, patients still have questions…and I have to end the session…then refer them to Clinical Two" | No recommendation provided; Solution 5: Educational Content via Mobile App |  |  |  |
