# Supplementary material for: Supporting Patients With Breast Cancer and Providers Through Treatment and Survivorship: Multimethod Implementation Study of the MyJourney Platform
Source: JMIR Cancer. 2026 Jun 10;12:e87973. doi: 10.2196/87973 (PMC13254169; doi:10.2196/87973)
Supplement: Multimedia Appendix 8 [file cancer-v12-e87973-s008.docx]

| **Challenge category: sub-category** | **Challenge [Role; PUID]** | **Illustrative Quote** | **Recommendation(s); MyJourney feature addressed (Yes/No)** | **Clinical context / Sub-phase** |  |  |  |
| --- | --- | --- | --- | --- | --- | --- | --- |
| **Health resource challenges** | | | | |  |  |  |
| **Human resources:** Limited staff availability | Some chemotherapy drugs have a high risk of patient reactions, requiring nurses to spend more time with those patients and impacting their ability to care for others [Nurse; P04] | *“Patients' flow is sometimes too much, and we have a lot of priorities…more patients per nurse, this is where the clinical concern comes in”* | No recommendation provided; No | During chemotherapy appointment |  |  |  |
|  | High patient flow leads to scheduling difficulties, clinical concerns, and potential patient safety issues [Nurse; P04] | -- | (i) Have additional staff, such as nurse practitioners, nurse navigators, or clinic nurses, to assist with patient follow-ups and continuous care; (ii) Designate a person to handle the volume of patient voicemails"; No |  |  |  |  |
|  | Nurses in the chemotherapy clinic experience limited human resources such as, clinic nurses or nurse practitioner [Nurse; P04] | *“We have more volume…And then, again, human resources itself are another big challenge because we have nurses who are not able to make it to work”* | No recommendation provided; No |  |  |  |  |
|  | Patients who walk into the clinic can be unpredictable and exhibit high anxiety, leading to frustration for both patients and nurses [Nurse; P01] | *“Walk-ins, [can be] very, high-anxiety patients. If I say no to them, are they going to understand? …First of all, they'll get frustrated. I'll get frustrated. Next time, they will not report something if it's important”* | No recommendation provided; Solution 5: Educational Content via Mobile App | Care coordination |  |  |  |
| **Human resources:** Division of responsibilities | Perception that the handover process for patient questions is not evenly distributed between nurses and pharmacists, placing additional pressure on Clin Two pharmacists to address these questions promptly [Pharmacist; P05] | -- | Implement a system or process that allows Clin Two pharmacists to seamlessly hand off remaining work tasks/questions to another pharmacist, to ensure an even distribution of workload; No | Additional counselling and support |  |  |  |
| **Physical space resources:** Limited space | Perception that nurses in the chemotherapy clinic do not have enough physical space to accommodate the volume of patients [Nurse; P04] | *“We have more volume, and we have less space”* | No recommendation provided; No | During chemotheraphy appointment |  |  |  |
|  | There is a lack of physical space at the chemo clinic and Day Medicine for patients to receive blood transfusions, often requiring them to be sent to the emergency department as a less ideal last resort [Nurse; P03] | *--* | No recommendation provided; No | Care coordination |  |  |  |
| **Quality of care challenges** | | | | |  |  |  |
| **Appointment:** In-clinic wait time | Delays in drug preparation and dosage confirmation by the pharmacy result in patients waiting longer in the clinic for their medications [Nurse; P01] | -- | Consider adopting a two-day model to reduce delays in drug preparation and shorten wait times for treatment; No | During chemotherapy appointment |  |  |  |
| **Patient education and counselling:** Information overload | Patients often feel frustrated or overwhelmed by the amount of information they receive on the day of their chemotherapy treatment [Nurse; P06] | *“The biggest challenge for the patient is it's a lot of information for them. They are overwhelmed…they don't process it well because it's a lot of information”* | No recommendation provided; Solution 5: Educational Content via Mobile App | During chemotherapy appointment |  |  |  |
| **Symptom management** | Follow-ups with patients who received after-hours care could be avoided with a proper initial assessment by the nurse providing the service [Nurse; P01] | *“[After-hours staff] often say liaise back with the with the oncology clinic in the morning, but they should be confident with their assessment...You're not instilling confidence in the patient”* | Establish an after-hours service at NYGH so nurses can call the patient and follow-up with them directly; No | After hours |  |  |  |
| **Continuity of care** | When a physician, nurse, or other healthcare professional receives a question from a patient for a pharmacist, and the patient is not on-site, the pharmacist can only respond by phone or in-person [Pharmacist; P05] | -- | Develop a secure platform, preferably a two-way system for communicating with patients after they have left the hospital while ensuring patient privacy and confidentiality; No | Additional counselling and support |  |  |  |
|  | Patients do not have 24/7 access to a clinician or pharmacist during after-hours, and the pharmacy department lacks the resources to provide continuous service [Pharmacist; P05] | "From a patient's perspective…pharmacist's advice accessible 24/7 would be valuable…But how do you staff that?" | The charge nurse should collect patient questions/reports in after-hours service for the pharmacy in one place that is easily accessible to pharmacists and can be addressed systematically; No | Additional counselling and support |  |  |  |
|  | The rotation of pharmacists at the chemo clinic results in a lack of a single point of contact for patients, making it difficult to build trust in pharmacy care [Pharmacist; P05] | *“I think the challenge is that we don't have a role that is specifically one person at any given time. It's desirable for the patient as well to know that they have a single point of contact. So, who's the pharmacy that's attached to them, right?”* | Assign one consistent point of contact pharmacist per patient throughout the patient's care; No | Additional counselling and support |  |  |  |
| **Administrative and process challenges** | | | | |  |  |  |
| **Communication challenges:** Limited communication delivery methods | The charge nurse can only relay after-hours questions to pharmacists verbally or via email, limiting communication effectiveness [Pharmacist; P05] | -- | The charge nurse should enter all after-hours questions and reports into a centralized system for systematic distribution, enhancing communication with pharmacists; Solution 1: Treatment Summary; Solution 3: Customized Notes, Tasks and Reminders for Users | Care coordination |  |  |  |
|  | The absence of a secure email system or a centralized repository of patient emails makes it difficult to communicate and share information with patients electronically [Pharmacist; P05] | "It is a bit challenging to provide the information as thoroughly as we'd like [to patients]. We don't have a mailing or communication system…So if we wanted to provide any type of websites, web links, or documentation, we must give it verbally, by phone, or have patients pick it up later" | While a two-way communication platform with patients would be ideal, staff resource limitations may make a one-way system more feasible; Solution 5: Educational Content via Mobile App | Additional counselling and support |  |  |  |
| **Incomplete information:** Delays | There are sometimes delays in physicians reviewing patient results and providing guidance on next steps, causing extended waiting times for patients [Nurse; P01] | -- | No recommendation provided; No | Care coordination |  |  |  |
| **Incomplete information:** Not up to date | Clin Two pharmacist returns an after-hours call unaware that the patient has already been followed up, leading to unnecessary duplication [Pharmacist; P07] | -- | No recommendation provided; Solution 1: Treatment Summary;  Solution 2: Upcoming / Past Appointments View; Solution 3: Customized Notes, Tasks and Reminders for Users | Additional counselling and support |  |  |  |
| **Notification challenges:** Lack of notifications | Nurses do not receive notifications about upcoming appointments or changes to patient schedules and must rely on secretaries or personal tracking methods to stay informed [Nurse; P01] | "I'm always writing stickies all over the place, and I've got a book now, it's a calendar that I write things down in" | No recommendation provided; Solution 2: Upcoming/Past Appointments View;  Solution 6: Overview of Bloodwork Appointments and Test Results | Care coordination |  |  |  |
|  | Nurses frequently check PowerChart for CT scan scheduling updates due to the lack of notifications [Nurse; P01] | *“For PowerChart, I check 100 times in a day [because there are no notifications]”* | No recommendation provided; Solution 6: Overview of Bloodwork Appointments and Test Results | Care coordination |  |  |  |
|  | The patient MRN must be manually entered into Cerner PowerChart to verify if a patient has checked in for their CT scan appointment, as the system does not provide notifications [Nurse; P01] | "I really wish that there was a thing that said, ‘Hey, this person checked in’…I don't want a notification on email…But just something…‘Oh, Mr. so-and-so checked into the emerg. Oh, look, he has a CT’" | Implement a notification system in Cerner PowerChart to alert nurses when patients check in for CT scans; Solution 6: Overview of Bloodwork Appointments and Test Results | Care coordination |  |  |  |
| **Workload management:** Manual processes and preparation | The process of documenting patient information, reviewing lab work, and following up with patients requires nurses to use various methods, such as sticky notes and calendar books, making it cumbersome to manage everything [Nurse; P01] | *“I'm always writing stickies all over the place. I've got a book now, but it's a calendar that I write things down in, and who am I supposed to call? What am I supposed to do the next day? So just say before, a patient used to have to do blood work, I don't know, in two weeks’ time. We got to just check their markers.”* | No recommendation provided; Solution 4: Tasks Reminders for Users on a Patient Level; Solution 6: Overview of Bloodwork Appointments and Test Results | Care coordination |  |  |  |
|  | Nurses often rely on personal methods (e.g., notebooks) to manage and track patient appointments, upcoming tasks, and documentation, which can be cumbersome [Nurse; P01] | -- | Implement a matrix or notification system to remind nurses about patient blood work or pathology results; Solution 3: Customized Notes, Tasks and Reminders for Users; Solution 4: Tasks Reminders for Users on a Patient Level; Solution 6: Overview of Bloodwork Appointments and Test Results | Care coordination |  |  |  |
| **Workload management:** Task volume and variability | Managing all patient calls each day while attending to other responsibilities is time-consuming for nurses [Nurse; P03] | -- | Have a symptom management nurse who can work daily to manage phone calls from patients; No | Care coordination |  |  |  |
|  | Manually documenting approximately 15-20 after-hours daily reports in PowerChart is both time-consuming and workload-intensive [Nurse; P03] | *--* | No recommendation provided; No | Care coordination |  |  |  |
| **Workload management:** Manual processes and preparation | Booking counseling appointments for patients transitioning between treatment regimens requires a manual process involving a paper sheet [Pharmacist; P02] | -- | Automate the scheduling process for counseling appointments, particularly when patients transition between treatment regimens; No | Additional counselling and support |  |  |  |
| **Health information technology system challenges** | | | | |  |  |  |
| **OPUS:** Manual entry of physicians’ orders | Entering written orders from physicians into the system is a time-consuming task for nurses [Nurse; P02] | "OPUS…because it's not interfaced with Cerner, we have to transcribe orders…There is a risk for errors…you could accidentally put a wrong dose" | Consolidate all necessary information for nurses into one program and allow electronic entry of physicians' orders; Solution 1: Treatment Summary | Care coordination |  |  |  |
|  | Nurses must switch between multiple programs to access different types of information in the current systems, complicating workflow [Nurse; P03] | *--* | Consolidate all necessary information for nurses into one program and allow electronic entry of physicians' orders; No | Care coordination |  |  |  |
| **Cerner PowerChart:** Patient data inaccessible | When documenting the need for a patient's blood work to be repeated as an interprofessional note in Cerner PowerChart, the note sometimes gets lost when the patient is admitted to the hospital [Nurse; P01] | -- | Have an interprofessional note feature in Cerner PowerChart specifically for oncology notes, making it easily accessible to all healthcare professionals and save time searching through records; Solution 3: Customized Notes, Tasks and Reminders for Users | Care coordination |  |  |  |
| **Cerner PowerChart:** Non-use by the outpatient pharmacists | Outpatient pharmacists, due to limited time or familiarity, often rely on phone calls and word-of-mouth communication rather than accessing information in Cerner PowerChart [Pharmacist; P05] | "The outpatient pharmacy…relies on phone calls…even though sometimes the information is right in front of them" | No recommendation provided; No | Additional counselling and support |  |  |  |
| **Limited system interoperability** | The nurses in the chemo clinic must navigate between the two systems, Opus and Cerner, since they are not connected with each other [Nurse; P04] | "We are working with two systems, OPUS and Cerner, and they don't connect…The nurses have to check both systems and use paper" | No recommendation provided; Solution 1: Treatment Summary;  Solution 6: Overview of Bloodwork Appointments and Test Results | Care coordination |  |  |  |
