# Supplementary material for: Supporting Patients With Breast Cancer and Providers Through Treatment and Survivorship: Multimethod Implementation Study of the MyJourney Platform
Source: JMIR Cancer. 2026 Jun 10;12:e87973. doi: 10.2196/87973 (PMC13254169; doi:10.2196/87973)
Supplement: Multimedia Appendix 9 [file cancer-v12-e87973-s009.docx]

| **Challenge category and sub-category** | **Challenge [Role; PUID]** | **Illustrative Quote** | **Recommendation(s); MyJourney feature addressed? (Yes/No)** | **Clinical context / Sub-phase** |
| --- | --- | --- | --- | --- |
| **Health information technology system challenges** | | | | |
| **Community Care Access Centre (CCAC):** Lack of central CCAC system | It is difficult navigating the Community Care Access Center (CCAC) systems since they are all in different regions [Nurse; P01] | "It's not comprehensive. Data accessibility depends on where the patient lives. It doesn’t list supplements, OTC medications like Tylenol,…or prescriptions paid for privately because the government doesn’t cover them" | No recommendation provided; No | During chemotherapy appointment |
