# Supplementary material for: Supporting Patients With Breast Cancer and Providers Through Treatment and Survivorship: Multimethod Implementation Study of the MyJourney Platform
Source: JMIR Cancer. 2026 Jun 10;12:e87973. doi: 10.2196/87973 (PMC13254169; doi:10.2196/87973)
Supplement: Multimedia Appendix 10 [file cancer-v12-e87973-s010.docx]

| **Domain** | **Chemotherapy Clinic** | **Breast Diagnostic Clinic** | **Representative Quotes** |
| --- | --- | --- | --- |
| **Usability** | 1.5 weeks: SUS 76.3 (SD=8.8), excellent usability  6 weeks: SUS 86.3 (SD=1.8), best imaginable usability | SUS 81.3 (SD=8.8), excellent usability | *“I think it’s... it’s an easy program. I use it every day... I like it… It’s easy on the eyes”* (P01, Chemo Clinic) |
| **Usefulness** | 1.5 weeks: TAM 3.9/5 (SD = 0.4)  6 weeks: TAM 4/5 (SD = 0) | TAM 3.9/5 (SD = 0.3) | “For me, it’s about having fewer places to look… it helps me do my job better and save time” (P09, BDC) |
| **Ease of use** | 1.5 weeks: TAM 4.3/5 (SD = 0.3)  6 weeks: TAM 4.8/5 (SD = 0.3) | TAM 4/5 (SD = 0.4) | “It’s very easy to use and organized… instead of clicking or going to a million places in Cerner, it’s right there” (P01, Chemo Clinic) |
| **Overall satisfaction** | 1.5 weeks: Scale of not at all satisfied to very satisfied, response of satisfied  6 weeks: Range of satisfied–very satisfied. | Range of satisfied–very satisfied | “Highly positive… I prefer MyJourney to Cerner” (P01, Chemo Clinic) |
| **Perceived benefits** | Centralized info, snapshot of treatments, fewer personal tracking methods, responsive support, improved care team communication, very easy to learn how to use | Saves time, reduces manual entry, one-stop search for patient info, supports following patients across stages, responsive support | - *“The benefits are, as I said, it’s everything is right there and... you’re able to see a snapshot”* (P01, Chemo Clinic) - *“I like the fact that you can write a little note... you’re able to see a snapshot of the conversation you had. I can also create tasks for myself, and it’ll send me reminders”* (P01, Chemo Clinic) - *“I'm able to reach out to the team for any technical issues, questions or concerns that I may have”* (P01, Chemo Clinic) - *“Before I used to have to check Cerner and SharePoint... Now a lot of times I can just look in one place in MyJourney”* (P09, BDC) - *“If a doctor calls to check on a referral, I can look up the patient and confirm if they’re booked... I use it like a search engine for referrals”* (P09, BDC) - *“Those notifications are extremely important... They’ve reduced the time I spend checking reports”* (P10, BDC) - *“It allows many of us to see the status of a patient and where they are in the system at any given time”* (P10, BDC) |
| **Challenges** | \| Reminders visible only in-app; lack of pathology/bloodwork integration; limited customization; instillation to computers occurred during work hours, impeding their workflow \| \| --- \| | Glitches (missing or outdated appointments), inconsistent notifications, no document attachment option | - *“What would be really good though is... if like there’s a pathology tab, if you just double click onto that, it’ll take you to the Cerner pathology or like the pathology would be uploaded there”* (P01, Chemo Clinic) - “Sometimes the appointment doesn’t show up, or it’s missing altogether” (P09, BDC) - *“Sometimes I don’t get notified, or the screen looks outdated until I refresh it... It’s not always consistent yet”* (P10, BDC) |
| **Recommendations** | Add email notifications, expand system integration, enable customizable lists, improve installation timing, broaden scope to all cancer patients | Add accountability features, allow document uploads, digitize paper charts, integrate with MCC rounds | - *“So, I’ve got four physicians, and I wish that I was able to filter my patients and have like a [personal] list... versus me…seeing other patients”* (P01, Chemo Clinic) - *“I wish it was done [implementation on computer] sort of after-hours versus during hours because it was impeding my work”* (P01, Chemo Clinic) - *“I wish like other cancers came on board, it would be easier”* (P01, Chemo Clinic) - *“We send letters to the family doctor, so it’s helpful to have those as a reference”* (P09, BDC) - “There should be accountability… we should be able to see who’s last in touching that patient.” (P10, BDC) |
| **Overall impressions** | Strongly positive, feasible for long-term use, expected to improve care transitions | Described as a “communication tool” that saves time, prevents missed patients, and is preferred over other systems | - *“For me, it’s about having fewer places to look for information... it helps me do my job better and save time”* (P09, BDC) - “It’s much better now and will only get better.” (P10, BDC) - *“It’s a communication tool, a means of ensuring patients aren’t missed... I think it’s a positive and it will be utilized quite well”* (P10, BDC) |

*SUS = system usability scale; TAM = technology acceptance model; BDC = breast diagnostic centre.
